# Enjoy Life LocallY (ELLY): A qualitative study of a co-designed healthy weight and wellbeing intervention in two disadvantaged Scottish communities

**DOI:** 10.1371/journal.pone.0361989

**Published:** 2026-11-05

**Authors:** Julie Cowie, Rhonda Archibald, Scott Findlay, Danielle Hutcheon, Sinéad Currie, Louise Hay, Elizabeth Cook

**Affiliations:** 1 Yunus Centre for Social Business and Health, Glasgow Caledonian University, Glasgow, United Kingdom; 2 NHS Forth Valley (Falkirk Health and Social Care Partnership), Scotland, United Kingdom; 3 The University of the West of Scotland, Scotland, United Kingdom; 4 Psychology, University of Stirling, Scotland, United Kingdom; 5 Denny Community Support Group, Denny, Scotland, United Kingdom; 6 Westfield Community Centre, Falkirk, Scotland, United Kingdom; UKM: Universiti Kebangsaan Malaysia, MALAYSIA

## Abstract

People who are unable to achieve a healthy lifestyle are at greater risk of type II diabetes, some cancers, heart disease, stroke, mobility problems and poor outcomes from disease. For individuals living in disadvantaged areas, life circumstances such as low income, unemployment, working anti-social hours and limited local services present barriers to the challenge of living healthy lives. Working with two disadvantaged communities in Forth Valley, Scotland, we co-designed and implemented an evidence-informed, incentive-based intervention, Enjoy Life LocallY (ELLY), to support citizens with healthy weight and wellbeing. The intervention comprised free soup twice weekly (café/delivery/pickup); a loyalty card (stamped upon engagement in community assets such as local activities, groups and clubs) exchanged for a £25 shopping card when a minimum of 9 assets over 12 weeks were attended; goal setting; information resources; and self-monitoring of weight and wellbeing. ELLY was delivered by volunteers over a 12-week period, from September 2023 to November 2023, to 75 residents. Qualitative interviews were conducted at 12-weeks with 10 participants per community, to assess study feasibility and conceptualise pathways to how ELLY led to healthy weight and wellbeing outcomes. Data were analysed using framework analysis. The ELLY intervention demonstrated strong acceptability and feasibility and was positively received by participants. In-depth interview data informed the construction of a conceptual framework identifying pathways through which the intervention supports healthy weight and wellbeing, including positive changes in behaviour, strengthened social connectedness, improved dietary habits, measurable participant-reported health improvements and the acquisition of new skills and knowledge. Collectively, findings support the hypothesis that engagement in the ELLY intervention was associated with a range of important health outcomes for participants, making a valuable contribution to the evidence base on community-based interventions and highlighting the important role of co-designed community interventions in supporting underserved, disadvantaged communities in health improvement.

## Introduction

The U.K. Office for National Statistics data show people living in disadvantaged areas are dying younger through increased risk of obesity-related conditions including diabetes, heart disease, some cancers and infections [1]. In Scotland, two-thirds of adults are overweight with a third living with obesity [2]. The root causes of obesity are complex and can be genetic, psychological, sociocultural, economic and environmental [3]. People living in areas of disadvantage often face barriers to achieving a healthy weight such as food insecurity, poor access to support services and physical activity opportunities, and less disposable income for food and leisure [4]. The prevalence of obesity risk factors such as over consuming high fat, high sugar food and drinks, and high levels of stress and trauma cluster within communities living in areas of disadvantage and can have adverse consequences throughout the life-course [5].

Many interventions which focus on healthy weight and wellbeing are effective for those who engage, but tend to attract more advantaged, resource and time-rich people who experience fewer barriers to involvement, potentially contributing to further health inequalities [6,7]. Consequently, innovative, cost-effective interventions are needed to engage and enable people living in disadvantaged communities to achieve sustainable healthy lives [7]. Systematic review evidence demonstrates that community engagement and intervention co-design can have a positive impact on health behaviours and health consequences [4,8], and has the potential to promote a sense of empowerment, self-efficacy and motivation for participants to engage in interventions [9]. However, involvement of target populations in the co-design of interventions supporting health has been limited, with a lack of process evaluation and outcomes [6,8], as well as inconsistent approaches to community engagement and empowerment [9]. There is a strong rationale for “putting the public back into public health” as a means of reducing health inequalities through community-led interventions; however significant gaps in the evidence base remain around the effectiveness of such approaches for challenges such as obesity and wider health inequalities [10].

Weight stigma is regarded as a significant barrier to achieving healthy weight, and rather than it motivating individuals to make positive behaviour change, has been shown to lead to adoption of maladaptive coping strategies such as disengagement with healthcare, eating disorders and reduced physical activity [11]. Interventions addressing holistic health, are not weight-centric but weight-inclusive, have greater potential to be impactful and reduce the risk of psychological consequences such as depression and anxiety [10–12]

The combination of incentive systems and community-led interventions represent a promising area of work, and has demonstrated an ability to motivate, engage and enable people living in disadvantaged areas to change behaviour [13,14]. Systematic review evidence highlights the effectiveness of financial incentives in achieving goals around healthy living and encouraging engagement in physical exercise and weight control [15]. In communities that face disadvantage, where intrinsic motivation to pursue health goals can be undermined by chronic conditions and limited agency and support, incentive-based approaches can act as a meaningful mechanism for enabling positive behaviour change. Community-based, co-designed approaches complement this by demonstrating capacity to generate innovative, locally based, tailored solutions to health-related behaviour challenges [6,10]. A recent review found that over half of community-based interventions implemented in disadvantaged communities led to improvement in at least one health behaviour. Despite this, the potential of upstream community approaches for reducing health inequalities remains insufficiently recognised and explored [16].

Addressing this evidence gap, the ELLY (Enjoy Life LocallY) study sought to co-design an incentive-based, community intervention to support healthy weight and wellbeing for those living in areas of disadvantage. Working in collaboration with two disparate disadvantaged communities in Forth Valley, Scotland, we co-designed and feasibility tested a novel evidence-informed incentive system to support community healthy weight and wellbeing. The study was conducted in two phases: co-design of the ELLY intervention through consolidation of systematic review evidence and empirical work with the chosen communities, and feasibility testing of the resulting ELLY intervention. [17] This paper presents the qualitative findings from the feasibility component of this work.

### Intervention and theoretical basis

The ELLY intervention is a place-based incentive system that acts as a connector to existing community assets (such as community clubs, groups, activities) to support citizens with healthy weight and wellbeing. The intervention is not prescriptive, but promotes autonomy, and puts emphasis on social cohesion, connectedness and relationships, and the influence these have on helping people to live well. The ELLY intervention builds on existing evidence from previous work team members have undertaken around the use of financial incentives, preparatory behaviours and participatory action research [18,19].

The intervention was co-designed with two disadvantaged communities in Forth Valley (Community C1, Community C2) and delivered over a 12-week period (September – November 2023). The intervention comprised: (i) Free soup twice weekly (sit-in café/delivery to home/pickup); (ii) Loyalty card and community asset engagement. The intervention featured a loyalty card which was stamped weekly for engagement in local community assets to encourage preparatory behaviours towards key study outcomes around weight, wellbeing and social connectedness. Community assets that agreed to be part of the ELLY intervention were broad and inclusive, consisting of arts and crafts, physical activity, nutrition-focused classes, and social groups. Assets were usually free to attend, with only 1/22 and 6/24 activities charging a small fee in communities C1 and C2 respectively. Participants who achieved 9 stamps on their loyalty card (equating to attending at least 1 activity, per week, over 9 out of 12 weeks of the ELLY intervention) were rewarded with a £25 shopping card at 12-weeks; (iii) Goal setting. At the baseline appointment, participants were informed about the optional aspect of goal setting for ‘living well’. Participants were given the opportunity to set (outcome or behaviour) goals relating to weight, wellbeing and other personal priorities. Goal setting was participant driven however the researcher encouraged generation of SMART (Specific, Measurable, Achievable, Relevant, and Time-bound) goals to achieve over the duration of the intervention. No specific action plans were developed, however, the researcher signposted participants to the other intervention components and community assets to support participants in achieving goals. Goals were reviewed at 12-week appointments; (iv) Website/written materials. The ELLY website and written handouts provided local asset/activity ‘*What’s on’* information and optional self-monitoring of weight and wellbeing was available via the website. Further details of the intervention described using the Template for Intervention Description and Replication (TIDieR) Checklist [20] are provided in Fig 1.

**Fig 1:**
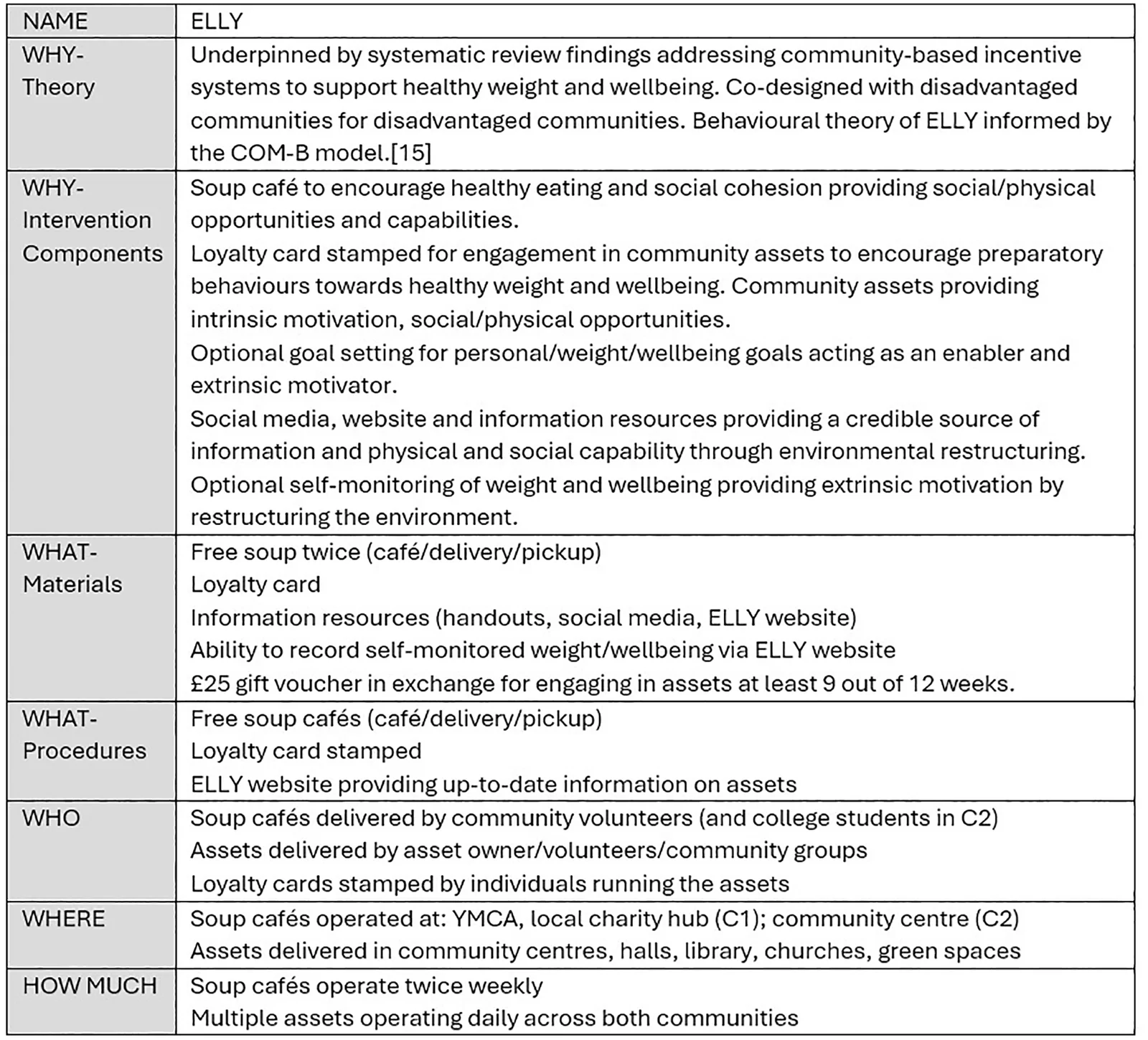
TIDieR checklist for the ELLY intervention.

## Materials and methods

The study design was a single arm, prospective intervention feasibility study. Setting: Two disadvantaged communities (Scottish Index of Multiple Deprivation (SIMD) 1–3) in Scotland were selected, one in a large rural area with small town centre (population ~ 1.5K) (C1), the other a small and urban community (population ~ 9K) (C2), enabling contextual comparison. The target area within each community was within a 20-minute walk to main community assets. Community 1 (C1) SIMD levels ranged from 1–3 (quintile) in the target area, with less-disadvantaged areas (SIMD 4–5) on the outskirts of the community. Accommodation in the C1 target area was predominantly public (social) housing. Community co-applicants operated voluntary organisations in two different sides of the target area and had not previously interacted. Local assets were predominantly based at community hubs, the local library, a community hall and the local church. The largest supermarket was a 10-minute walk from the town centre with the alternative being smaller, local shops. Community 2 (C2) SIMD levels ranged from 1–2 (quintile) and was predominantly public (social) housing. Local assets were mainly based at the community centre operated by a community partner. A retail park (and the closest supermarket) was a 20-minute walk away with a small grocery shop and petrol station located in the target area.

The inclusion criteria for research participants were any adult aged 18 or over living within 20-minute walking distance from main community assets. The exclusion criteria were an inability to understand information and consent, and/or not planning to reside in community for the duration of the intervention.

Self-reported outcome data around healthy weight and wellbeing were collected using validated and adapted questionnaires focusing on wellbeing (WEMWBS), quality of life (EQ-5D-5L), social connectedness [21], lifestyle and NHS use. Adapted questionnaire design was informed by community consultation. Outcome data was collected at baseline and 12 weeks via individual appointments with participants in community settings or at a participants’ home.

Ethical approval was granted from Glasgow Caledonian Ethics committee (Nursing and Community Health Research Ethics Committee (REC), HLS/NCH/22/036). Written informed consent was obtained by all participants prior to taking part in the study. Participants consisted of adults residing in the two target communities.

### Qualitative data collection

Recruitment to the study took place between 20^th^ June 2023 and 31^st^ August 2023. A range of recruitment strategies were used, including engagement through community assets, voluntary organisations, pop-up cafés and flyers circulated to local schools and businesses. We aimed to recruit a minimum of 30 adult participants per community to the ELLY intervention, and 10 of these participants, per community, for interview. The qualitative component of the study adopted an interpretivist qualitative descriptive approach where semi-structured interviews were used to explore participants’ experiences of the ELLY intervention. This component is reported according to the COREQ checklist [22] in S1 Appendix. Purposive sampling for interviews was informed by baseline participant characteristics and informal feedback from intervention volunteers relating to diversity of participants’ socio-demographic characteristics (age, sex, employment status, household composition), BMI at baseline, engagement in the intervention over the 12 weeks, and goal setting conducted at baseline. The pragmatic, predefined number of interviews was in line with the feasibility nature of the study and consistent with recommendations in the literature where the principal purpose of the qualitative data is to inform intervention design rather than achieve data saturation [23]. Participants were invited to share their ELLY experience through semi-structured interviews conducted at 12 weeks post-start of the intervention. While questions and prompts were used, themes were also explored in a narrative style to enable flexible exploration of health and wellbeing topics and allow any emerging themes to be explored in greater depth [24]. Participants were asked to tell their ELLY story, share their views about different components of the ELLY intervention, share experiences of attending the assets (activities, groups and clubs) that they engaged with, and reflect on any ways in which involvement in ELLY had impacted their health and wellbeing or behaviours.

Before commencing an interview, participants were provided with an information sheet and consent form and given a minimum of 48 hours to decide if they would like to take part. Participants were also assured of their anonymity and their right to withdraw at any point of the interview, without reason. The information sheet provided a lay language summary of the study and what taking part would entail, alongside details of safeguarding procedures, management of sensitive disclosures and signposting to appropriate support services should participants wish to access them. Depending on participant preference, interviews were conducted at home or in community settings (e.g., library, community centre) and were all face-to-face. All interviews followed a pre-defined topic guide (see S2 Appendix), lasted approximately 30 minutes and were audio recorded using an encrypted Dictaphone, then transferred to a password encrypted computer folder. All interviews were conducted by two project researchers (SF, RA), who were both known to participants, but were not involved in delivery of the intervention. Researcher field notes were taken at all interviews and used to inform the qualitative analysis. Discussion of these notes and weekly meetings of fieldwork researchers with the wider study team supported reflexivity and researchers’ awareness of positionality. Meetings facilitated discussion around how data were interpreted, underlying assumptions, and how individual perspectives and opinions may shape the findings.

### Data analysis and development of conceptual logic model

NVivo 12 software was used to support analysis of the qualitative data arising from interviews. An initial coding framework was independently developed by two researchers (DH, RA) after reviewing a diverse sample of five interviews. This was followed by team discussions to refine and finalise the coding framework and to identify key themes. Each theme was then examined in greater depth and further divided into subthemes, with overlapping or duplicate codes combined. The analysis was led by one researcher (DH), with 10% of the data independently cross-checked by a second researcher (RA). Any disagreements were resolved through discussion with a third researcher (SF). Coding and interpretation were regularly reviewed by the principal investigator (JC). Researcher field notes were also used to support interpretation of the interview data. Emerging themes were reviewed during weekly team meetings. DH had not been involved in any other aspect of the project. Her participation in the qualitative coding added to the rigour and impartiality of the analysis.

A conceptual logic model was developed through a systematic and iterative process involving the wider team, exploring interview data that described intervention components, mechanisms and processes that led to health and wellbeing outcomes, and the links between them. Having formalised initial models, the components were explored in terms of how they functioned, and behaviour change elements underpinning them were identified using the COM-B model [25]. Utilising the COM-B allowed components to be categorised as provision of motivation (e.g., incentivisation provided by the loyalty card), opportunities (e.g., environmental restructuring such as provision of the café) and capability (e.g.,e.g., training of volunteers).

## Results

### Participant demographics

Target recruitment to the feasibility study was met, with 35 and 40 adult participants recruited in communities C1 and C2 respectively. A total of 20 participants (10 per community) were asked to take part in interviews, and all agreed. Table 1 reports baseline characteristics of the interview participants. The mean average age of participants was 51 (SD = 13.1), with BMI of 33 (SD = 6.6). 14/20 (70%) of participants were female and 17 (85%) lived in disadvantaged areas (as defined by SIMD quintiles 1–3). Co-morbidities were reported by 12 (60%) participants, 15 (75%) were ethnic group white, and 15 (75%) lived with one or more person. The average household size of participants was 2.8. The interviewees comprised retirees (8(40%)), individuals not in employment due to long-term sickness or disability or an unstated reason (6(30%)) and individuals in employment (6(30%)). These statistics are representative of the wider population of ELLY feasibility study participants.

**Table 1:** Demographic information.

ID^a^	Age	Gender	Ethnic group	Employment status	Health status	SIMD
C1, P2	41	Male	Asian or Asian British	Retired	High blood pressure, heart condition, diabetes	2
C1, P19	50	Female	White	Not in employment for other reason	No disclosed health conditions	3
C1, P24	38	Female	White	Full time employment	No disclosed health conditions	3
C1, P26	38	Female	White	Other (not disclosed)	No disclosed health conditions	4
C1, P30	38	Female	White	Not in employment due to long term illness or disability	Mental health condition	2
C1, P31	46	Female	White	Not in employment due to long term illness or disability	High blood pressure, mental health condition	2
C1, P32	70	Female	White	Retired	No disclosed health conditions	1
C1, P36	71	Female	White	Retired	Diabetes, cancer, arthritis	1
C1, P43	76	Male	White	Retired	High blood pressure, heart condition, diabetes, mental health condition	1
C1, P51	69	Female	White	Retired	High blood pressure, heart condition, diabetes, arthritis, mental health condition	3
C2, P3	39	Male	Black, African, Caribbean or Black British	Part time employment	No disclosed health conditions	1
C2, P19	68	Male	White	Retired	Heart condition	2
C2, P27	58	Female	White	Retired	Heart condition, arthritis, mental health condition	2
C2, P34	59	Female	White	Not in employment due to long term illness or disability	Heart condition, arthritis, mental health condition	3
C2, P40	50	Male	White	Not in employment due to long term illness or disability	Heart condition, arthritis, mental health condition	3
C2, P41	43	Male	Mixed or multiple ethnic groups	Full time employment	High blood pressure, cancer	5
C2, P101	47	Female	White	Retired	Mental health condition	1
C2, P105	40	Female	Asian or Asian British	Part time employment	No disclosed health conditions	2
C2, P120	46	Female	Other ethnic group	Other (not disclosed)	No disclosed health conditions	1
C2, P304	^b^	Female	White	Not in employment due to other reason	No disclosed health conditions	5

^a^C1 community 1, C2 community 2, P<number > = participant number; ordered by community number, participant number. ^b^ Not reported

### Outcomes of the ELLY intervention

Table 2 provides an overview of the main themes arising from the participant interviews. Detail of each theme together with participant quotes and corresponding visual diagrams that demonstrate logic model pathways are provided in subsequent sections, with further supportive quotes for each theme discussed provided in the S3 Appendix.

**Table 2:** Summary of key themes.

Theme	Description/ Summary	Component
Snowball recruitment	Participants spoke about actively encouraging family, friends and neighbours to take part in ELLY.	Mechanism
Connecting with community assets	Participants reported gaining new knowledge and awareness of community assets and feeling motivated to leave their house and engage with them.	Mechanism
Positive changes in behaviour	Participants reported adopting new positive habits and behaviours, as well as having an increased sense of purpose and achieving personal goals.	Outcome
Social connectedness	Participants reported that the intervention helped them interact with others more, develop new friendships, feel less isolated and feel connected to their community.	Outcome
Healthier diet	Participants felt the intervention had helped them make improvements in their diets and nutrition.	Outcome
Health improvements (mental and physical)	Participants reported taking part in the intervention had led to improved fitness, confidence, happiness, and better management of existing health conditions.	Outcome
New skills and knowledge	Participants reflected on new skills they had learnt (e.g., fixing bikes, crafting, supporting peers) whilst being part of the ELLY intervention, allowing them to socialise and integrate more into the community.	Outcome

#### Snowball recruitment.

Analysis of interview data revealed snowball recruitment as a notable intervention mechanism, where participants proactively recruited others in their community by sharing information about the study. This peer-to-peer spread of information not only ensured the intervention had wide ranging reach across communities but also reflected participants’ enthusiasm for being more involved in community activities, more socially interactive, and ultimately, being part of the ELLY intervention. Snowball recruiting was predominantly reported in community C1 and to a lesser extend in C2, potentially reflecting a comparatively lower sense of existing community cohesion in community C2.

*“I’d put a note through her door saying, why don’t you do this...Then after that she was maybe coming every other week... Then she’s invited other people” [C1, P26]*

A small number of C2 participants commented that being involved in ELLY had led them to bring their children along to activities too. Many participants had been snowball recruited themselves, finding out about ELLY from friends or family who were already participating in the programme.

*“…when she spoke about it, I thought do you know that’s a good idea, that will get me out for even just two hours. And I said to my neighbour, do you fancy going because I would say to her she needed to get out more, so she done the same” [C2, P51]*

This spread of information about the intervention through peer-to-peer networks reflects an important reach and dissemination pathway through which ELLY was perceived to operate, rather than a standalone outcome. This is depicted in Fig 2.

**Fig 2:**
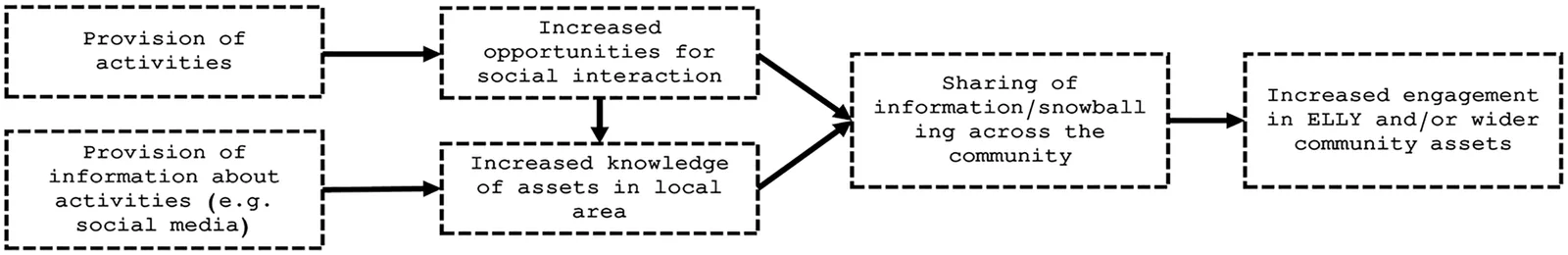
Snowball recruitment as a reach and dissemination mechanism.

#### Connecting with community assets.

New knowledge of community assets was acquired through a variety of ELLY resources, including the “What’s on in your community” ELLY handout, the study website, posts and interactions on social media, and hearing about other local assets through attendance at ELLY activities. Participants described having an increased awareness of assets and activities within their community and for many, this represented a positive shift in mind set about what their community had to offer. This translated into feeling more positive about where they lived, fostering a sense of pride and keenness to become more involved in the community.

*“It introduced me to other places here. I didn’t walk before in [name of park], and after the project I started to walk in [name of park] as well” [C2, P40]*

*“I think it has definitely helped to open my eyes to more of the things that are going on in the community. And I think probably what I will do from now on is try to be more proactive in finding out what’s happening” [C1, P19]*

Participants also reported getting out of the house more than they used to prior to the ELLY study. This was viewed as particularly significant for those who had struggled to leave their house due to personal or health-related challenges. ELLY activities were described as one of a few opportunities available for interaction with others:

*“…before [ELLY] it was a lot to get out of the house. I’ve changed big time for getting out of the house. I was stuck in the house all the time, with my depression and that. I’m out of the house every day” [C1, P31]*

Connecting and engaging with community assets is reflected in Fig 3, depicting the reported pathways by which this mechanism operated and the outcomes it contributed to.

**Fig 3:**
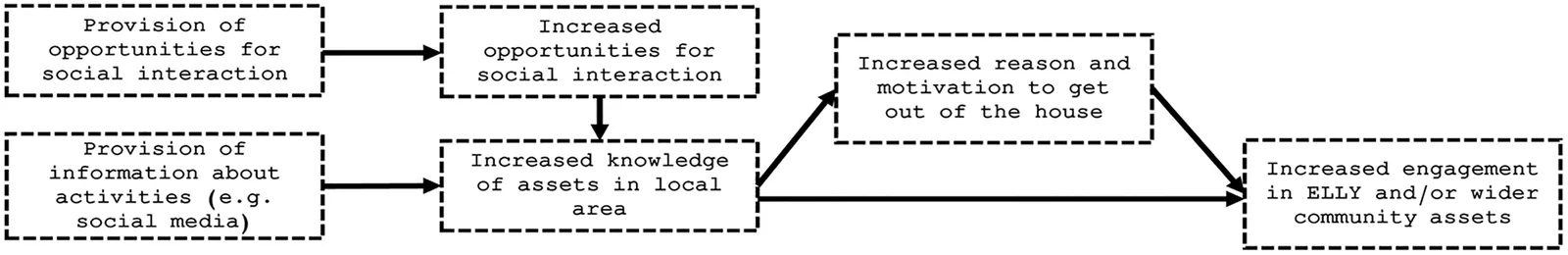
Connecting with community assets awareness and engagement mechanism.

#### Positive changes in behaviour.

A significant number of participants reflected upon their success with positively adapting habits and behaviours, with many participants selecting weight and wellbeing goals to focus on. Participants linked new behaviours and habits to becoming healthier:

*“I feel the ELLY Project was very helpful for me…I did a lot of activities, achieved my goal, which was my weight… Now I walk every night…I continue in this situation, like walking, and soup, and healthy food, that has helped me a lot.” [C2, P34]*

Some ELLY participants stated satisfaction with new behaviours as they felt these new habits gave them an increased sense of purpose and routine, which they linked to attending ELLY assets and working towards their goals.

*“I was made redundant…I kind of lost my self-esteem because I felt nobody wanted me with being made redundant… I was only going and having a shower once a week… Then [as a result of ELLY], I was having a shower every day, every night...it’s somewhere to be” [C2, P19]*

Hence, participants often linked the positive changes in behaviour to the goal setting component of ELLY and the provision of activities to support goal attainment. However, for a small number of participants, goals were not achieved, for example one participant shared *“I wanted to stop smoking, and I wanted to stop smoking weed. I failed dismally on both accounts” [C2, P27].* Despite not achieving goals, there was a recognition that the goal setting process was perceived to facilitate positive change towards where they eventually wanted to be, and the goals provided a means of measurement of progress:

*“My goal was losing weight and I didn’t hit it, well I’ve failed, no, I didn’t fail because I’ve grown towards where I need to be. So, it’s the idea of looking forward, of continued growth, of continuing growing…I think it helps people to refocus their own way of thinking of, well how do I want to move forward with my life and okay, I’m going to hit this point, now what.” [C2, P41]*

Participants reported that goal setting was found helpful in initiating behaviour change, but it was the intervention as a whole that was viewed as a key facilitator of sustained change. Participants also reported associated psychological benefits such as increased self-esteem which was frequently reported to support longer-term change.

A few participants reported they did not engage in goal setting and did not feel the idea was helpful with some reporting that goals just felt unattainable given their life circumstances. For example, one participant reflected that “*Goals wise I’ve not been very good…I’ve had a lot of stress over the last wee while between my son and my mum’s been in hospital for over a month now.” [C2, P304*], highlighting the need for goals to be appropriate, realistic and flexible given wider life circumstances.

Whilst the goal setting was perceived to initiate short- and long-term behaviour change in many participants, it did not benefit all, suggesting some degree of individual tailoring might be required in interventions such as ELLY in order to fully be effective for all participants.

Fig 4 depicts the different outcomes and behaviours participants felt they had achieved as a result of participating in the intervention.

**Fig 4:**
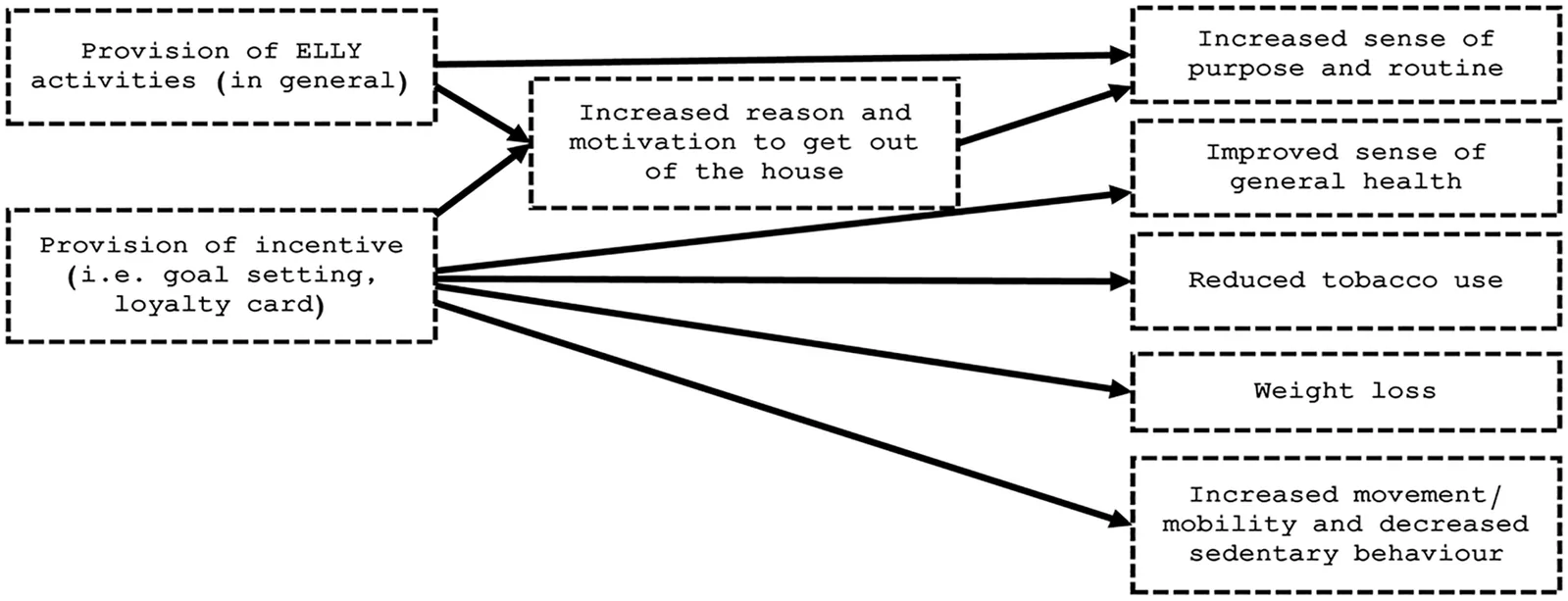
Positive changes in behaviour pathways to outcomes logic model.

#### Social Connectedness.

Many participants reported that being part of the ELLY intervention had increased their levels of social interaction and provided an opportunity to connect with others in the community:

*“It’s actually got me going out more, meeting other people, having a laugh, which is important… It’s been good, instead of sitting here vegetating in front of the telly” [C1, P36]*

Some participants felt taking part in the ELLY activities gave them a chance to meet new people of different ages and backgrounds that they normally wouldn’t have interacted with:

*“I think it’s a great way of forming bonds with people that you don’t mainly associate with, that you don’t normally run in the same circles with” [C1, P19]*

For some, increased social connection through the ELLY programme was found to increase their sense of belonging to their community. Participants also reported ELLY had provided opportunities for newcomers to the communities to become integrated and gain a sense of belonging:

*“…the community is growing and people are coming from different areas, but they have nobody, no friends, nothing…I think once they start coming into the group, they get to know each other. They start trusting in each other and then some people become close to some other people, make friends, become like a family which is good” [C2, P105]*

Participants from both communities felt ELLY was a catalyst for community connectedness. For example, one participant spoke fondly of the “lovely sense of community” [C1, P19] that ELLY facilitated and that they “...r*eally enjoyed the sense of community that was developing”*. This suggests that the intervention was important in providing an organic feeling of connection amongst participants, potentially acting as a means of social infrastructure facilitating new social connections and social capital through new and strengthened relationships.

The majority of participants from both communities stated that interacting with other participants and ELLY volunteers made them feel more socially supported. Social support was also experienced through peer networks, where participants helped each other in different ways:

*“…we have a WhatsApp group… It was encouraging each other. One of them is sending you a picture of her, “Oh, I done this many steps,” and like, “Oh, if she’s doing it, I’m doing it as well.” [C2, P105]*

Some ELLY participants stated they felt less socially isolated and lonely taking part in ELLY as it helped them gain confidence leaving the house and meeting new people. For example, one participant shared *“I’m very isolated… before (ELLY) I was very lonely, and I didn’t know anyone. During the project I got to meet new people and things and got new places to go to and new things to do rather than sitting in the house” [C1, P30].*

Whilst the majority of participants reported the intervention helped connect them to others, this was not true for all. Some participants reported that they did not quite fit with the groups offered and found integrating into the existing groups difficult*:*

*“I found some things very specifically orientated to different groups and I didn’t fit into those groups, so that makes it quite difficult … it’s probably a bit more daunting to approach a group which is obviously not your group…just to approach that sort of group at that point was a bit difficult.” [C1, P19]*

This was compounded by the irregularity of groups which sometimes hindered participants’ ability to connect with others:

“*I think they (activities) were very few and far between, so for example the [group name] project was only on once during this time because the first one was cancelled. And then they moved it to a different date, so during the time it was not as regular as I thought it might be and it’s difficult to build up relationships with people if you see them very infrequently.”* [C1, P19]

Such feelings of disconnect underscore the importance of ensuring community assets are flexible and responsive to community needs to maximise inclusivity, reach and relevance. The reported pathways to increased social connection outcomes are shown in Fig 5.

**Fig 5:**
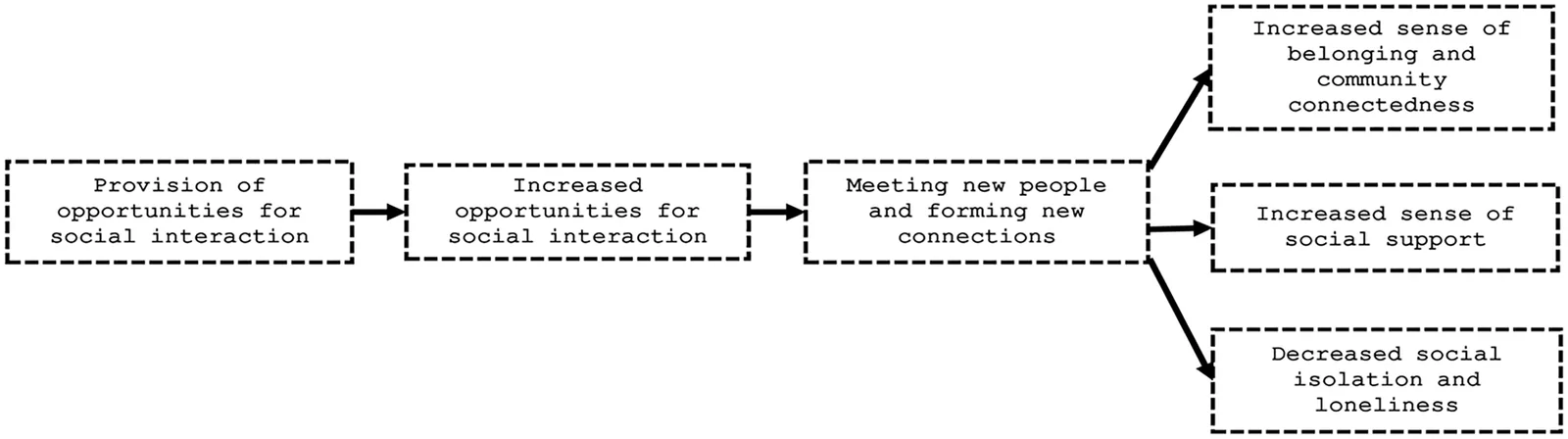
Social connectedness pathways to outcomes logic model.

#### Healthier diet.

An important theme arising from the data was that of perceived improved nutrition and a healthier diet. Some participants reported that participation in ELLY had improved their diet through access to healthier food and the opportunity to sample this for free:

*“[During ELLY] I was eating healthier, I was eating about two or three times veg a week, I had a vegan dinner once a week” [C2, P120]*

Discussions held between participants and ELLY volunteers at community assets also seemed to increase knowledge about heathier food options. In community C1, this was evidenced by volunteers at the soup cafés producing “make it yourself” soup cards in response to participants’ interest in making the soup at home as an affordable and nutritious meal option. Some community C2 participants, who had been diagnosed with physical and mental health conditions, also reported that being part of ELLY had ensured that they were gaining regular access to food and eating more appropriate portion sizes. These examples show ELLY’s perceived role in encouraging a change in behaviour from receiving and participating in provision of healthy food to motivating and enabling participants to engage in making their own nutritious food for themselves. This reported pathway from provision of healthy food through the ELLY cafés to potentially improving dietary intake is represented in Fig 6.

**Fig 6:**
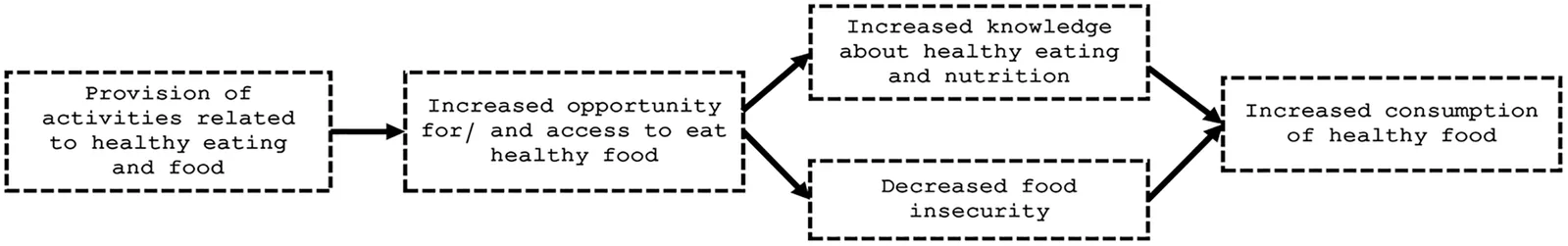
Healthier diet pathways to outcomes logic model.

#### Health improvements (mental and physical).

Many participants stated that through the physical activity-focused ELLY assets (or walking to and from activities), that they had engaged in more exercise and felt an increase in their physical fitness. By promoting local activities, it was reported that ELLY ensured participants could embed exercise into daily routines through walking to assets rather than having to use transport. For many, with time and budget limitations, this was seen to provide a means to increase exercise in a way that felt manageable, affordable and sustainable in the longer-term.

*“[As a result of ELLY] a barrier has been broken now…my mum and dad’s is quite a distance from here. I can walk very easily there, I can walk back, don’t need to take my car out as much…with the gym and stuff, I’m lifting again, which I was scared to do before but now because it’s increasing…it’s helping me a lot more.” [C1, P2]*

This reported physical health pathway is shown in Fig 7.

**Fig 7:**
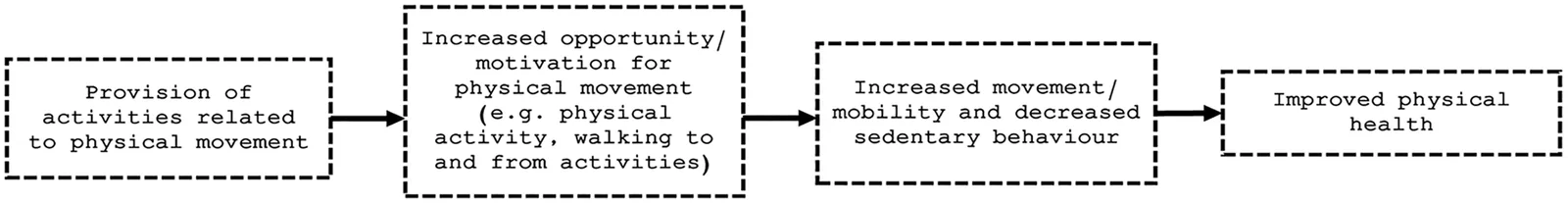
Improved physical health pathways to outcomes model.

Improved mental wellbeing was a significant theme that many participants discussed. A large proportion of participants from both communities reported that they felt being part of ELLY had increased their confidence to leave the house again and re-engage with others:

“…*it’s given me the confidence to go and join, after COVID, for me to walk into somewhere like that and meet complete strangers, it’s kind of, I won’t say it was a big deal but I was nervous about it but since then I’ve been fine and it’s given me the confidence now to go and join things” [C2, 19]*

Participants from both communities felt that taking part in ELLY had increased their overall happiness and made them feel more positive about their lives.

*“…as soon as I start walking, I feel more fresh. I’m more positive about things now. I thought, “It’s okay. It’s fine. This is life. Things happen”.…to walk and fresh air changes my thinking” [C2, P105]*

Some participants, who had disclosed diagnosed mental health conditions, perceived that ELLY had helped with their emotional wellbeing. Participants reported that they felt the opportunity to take part in the ELLY cafés, local activities and goal setting had helped them feel less anxious, more connected to others, and emotionally stable:

*“I was feeling not myself [pre-ELLY], I was feeling weak in myself basically. Today, I don’t feel that way. Today I feel a lot stronger and I’m trying to come off the majority of my medicines now that dealt with my anxiety and my depression…months back this wouldn’t have happened…this has helped because putting things into a goal format has helped me achieve them” [C1, P2]*

*“Before (ELLY) my time wouldn’t pass…through these activities, it flowed really nicely. For the moments which I used to have the anxiety in the morning, rather than watching a wall or looking outside, now it was a case of I had a reason to do something and during those times” [C1, P2]*

“…*there was a time when I was going a bit downhill…but as soon as I start walking, I feel more fresh…because if I don’t walk, I feel depressed” [C2, P105]*

The importance of the ELLY intervention being perceived to support good mental wellbeing cannot be understated. As evident from the interview data, perceived improved mental wellbeing led to participants feeling more confident, happier and less anxious which was reported to act as a foundation for participants’ willingness to try new things, become more socially engaged, and adopt healthier routines. These perceived pathways from ELLY components to positive mental health and wellbeing outcomes are represented in Fig 8.

**Fig 8:**
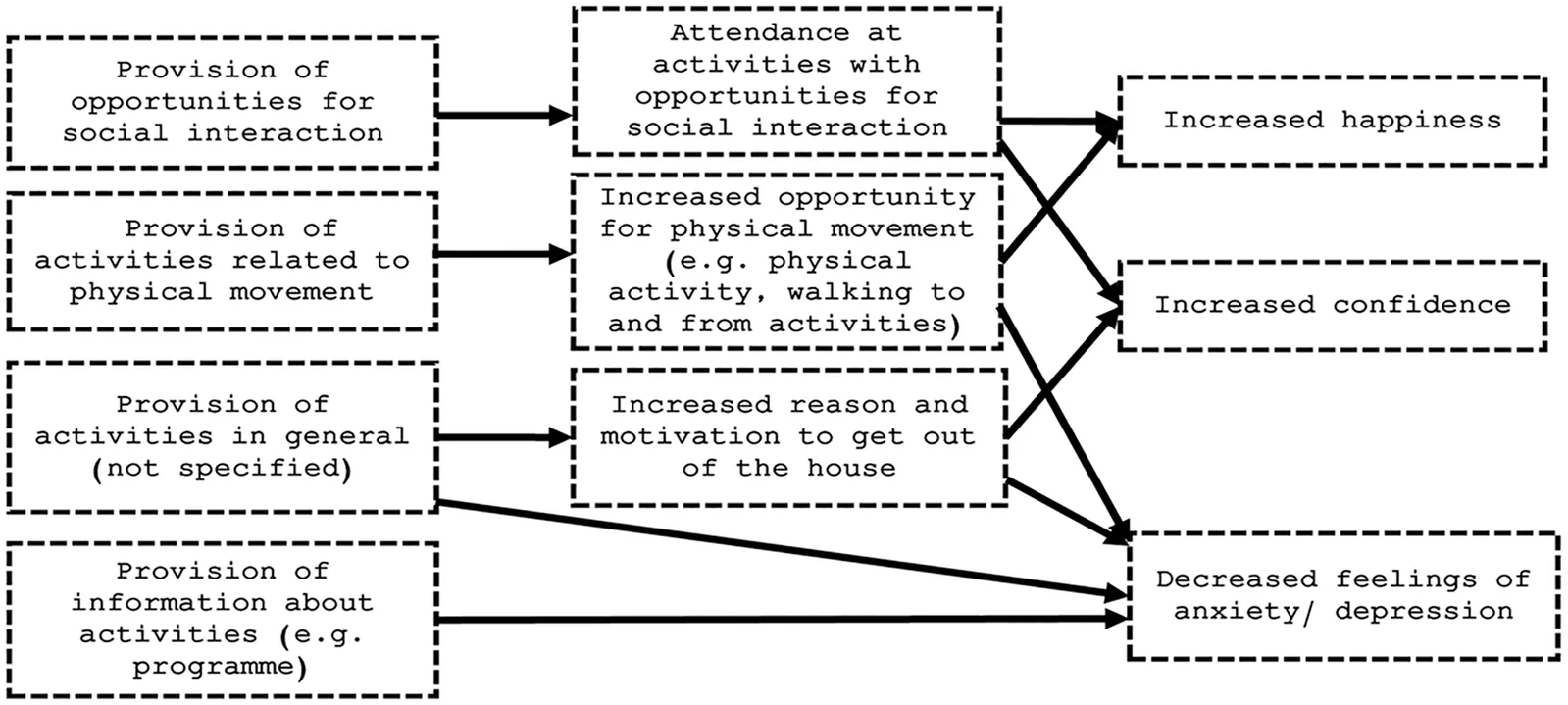
Improvements in mental health pathways to outcomes logic model.

#### New skills and knowledge.

Some participants felt ELLY had provided new opportunities to learn skills and knowledge related to specific activities. Participants described their enjoyment of learning new activities and challenging their usual daily routines to encompass something new. As a result, they felt they found new confidence and as sense of personal growth and development.

*“The [community voluntary organisation] have some activities that we partook in which would be something that I would never usually do…like, arts and crafty stuff, which was good” [C1, P24]*

*“I don’t normally take walks. That is not part of our culture. When we walk, we are walking to work or walking to do something. But walking here, I find out that walking is just an activity on its own. So, that is something new to me” [C2, P3]*

A small minority of participants reported they had not been made to feel welcome at the community groups they had attended and therefore were not able to engage and gain new skills or knowledge:

“*it just seemed unwelcoming. The organiser of the group didn’t feel, I felt anyway, he didn’t feel welcoming, they weren’t, I felt they didn’t want us there, you know, it felt kind of cliquish [C2, P19]*

The same participant, felt communication with them had been poor, sharing *“Nobody showed up the times I was here once. Nobody tried to contact me, find out what was wrong or anything like that. I’d left word that had been here for like 6:50 to 7:10. Again, nobody.” [C2, P19]*

A further participant reflected that joining a new group was just too daunting for them:

*“for me the walking into a place and not knowing anybody and take part in a group I would say was probably a barrier for me.” [C2, P304]*

Negative experiences around inclusion are unfortunate as they can reinforce existing feelings of social disconnect, anxiety and exclusion. The comments reinforce the need to identify and address potential barriers to engagement such as those highlighted in the interviews, to ensure mitigating measures can be actioned to promote better inclusivity and improve participants’ experience of engaging with such groups. The participant reported pathway for new skills and knowledge is shown in Fig 9.

**Fig 9:**
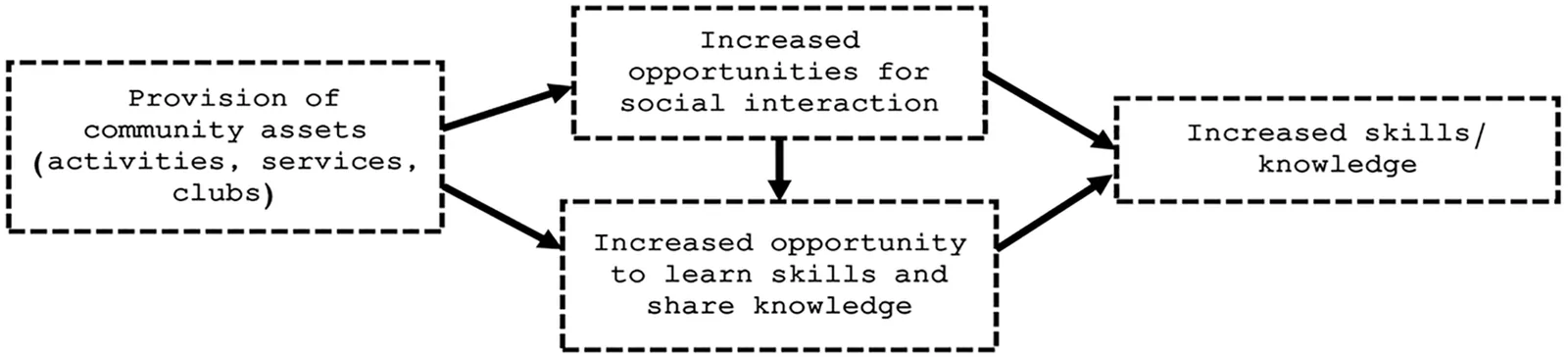
New skills and knowledge pathways to outcomes logic model.

### Pathway to outcomes logic model

Drawing on the findings above, participating in ELLY was linked to a broad range of positive outcomes for both participants and the wider communities. Changes in behaviour and associated outcomes were felt by participants to be driven by multiple interconnected mechanisms and therefore the individual logic models for each theme were synthesised into a single model to capture this complexity (Fig 10).

**Fig 10:**
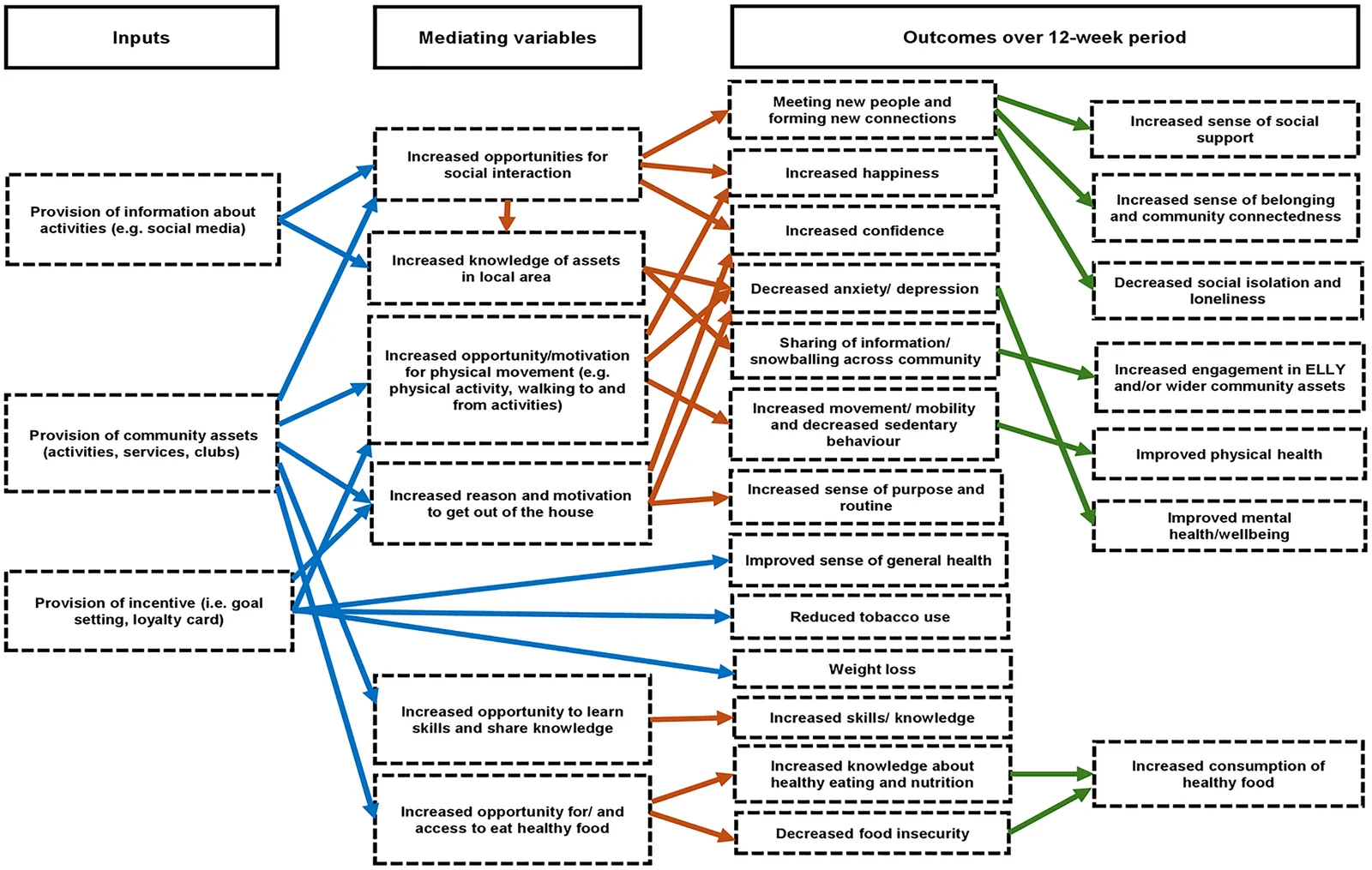
Synthesised conceptual logic model for all outcome pathways associated with participating in the ELLY intervention.

This logic model shows the perceived routes from ELLY inputs to outcomes were complex, multifaceted and interlinked, with participation seen to result in a range of differing effects on individuals and communities. The model also shows a degree of cross-over between outcomes. For instance, both opportunities for social interaction and the provision of information about activities were reported to have led to increased engagement in ELLY assets. As ELLY was a complex intervention the reported data did not always allow for clear causality between all intervention functions, behaviour change elements and related outcomes. The model distinguishes intervention mechanisms, such as snowball recruitment and community connections, from the outcomes they contributed to, such as social connectedness and positive behaviour change.

The conceptual logic model encapsulates pathways to outcomes observed for the duration of the ELLY intervention (12 weeks). This snapshot of participants’ experiences is valuable in understanding which immediate and intermediate outputs were observed but also determining how they were realised.

## Discussion

The aim of this study was to evaluate the feasibility of the ELLY intervention and explore qualitative interview data to understand perceived causal pathways by which the intervention supported participants’ healthy weight and wellbeing. Whilst this qualitative data alone is insufficient to establish definitive causation, which would require more in-depth mixed-methods research, it does offer rich and valuable insight into participants’ experiences and perceived pathways to observed outcomes.

Despite most participants choosing a weight goal, findings suggest that achieving a healthy weight was *not* perceived by participants as a motivating factor for changing behaviour. This aligns with existing literature around extrinsic motivations, such as striving for healthy weight, suggesting such motivations may prompt initial engagement with an intervention, but are unlikely to singularly lead to sustained behaviour change [26]. Rather, the wellbeing and social elements of ELLY were felt by participants to be the most motivating elements of the intervention, which is supported by existing evidence highlighting the importance of wellbeing as a driver for behaviour change in disadvantaged groups [18].

Quantitative outcomes of the study show changes in BMI, despite participants suggesting healthy weight was not the main reason for taking part in the intervention [17]. This suggests that ELLY potentially may reduce weight through preparatory behaviours associated with engagement in social activity. The findings suggest healthy weight may be understood as a “happy by-product” of increased wellbeing through engagement in community activities, better social connection, enhanced motivation, and healthier daily routines reported by ELLY intervention participants. This is consistent with previous research that demonstrates that social capital can positively or negatively influence body weight [27]. By focusing more on holistic wellbeing and strengthened social connections, community-based interventions such as ELLY may create a platform to support sustained behaviour change, with weight-related improvements occurring indirectly rather than being the main focus of an intervention.

Increased self-confidence and self-efficacy, which participants felt was as a result of taking part in ELLY appeared to act as a catalyst for further behaviour change for some participants. Since such transformations occur at a pace specific to each individual, a longer-term study is required to demonstrate possible positive behaviour change for more participants. For example, some participants reported they felt ELLY had improved their diet through access to healthier food; preparatory behaviour that supports a journey towards weight loss. In addition, others described being more active and motivated in other aspects of their lives, in addition to attending activities. A longitudinal study would establish whether such perceived changes in behaviour were temporary or sustained beyond the duration of the intervention.

Implementing the ELLY intervention in different communities with different contexts was seen to have significant impact on both how ELLY was received and delivered. Embedding ELLY within existing community structures was a key strength of the study and built local capacity and capability within the community and encouraged sustained use of the intervention and/or some of its components. However, variation in the willingness of community assets to take part in the ELLY intervention and differences in the expected support given by the research team during intervention delivery acted as barriers to the effectiveness of ELLY. This exemplifies the requirement for sustained transparency, trust and good communication in co-design processes [7]. Further, assessing community capacity and readiness in greater depth at the outset of the research would be prudent for future studies.

Literature has highlighted a need to investigate the specific impacts of co-designing interventions with communities and the use of empowerment-based approaches, especially for weight management and obesity-based activity [7–9] Participants in our study reported that embedding activity in local physical and social structures increased their engagement and sense of belonging, and the co-design aspect was found to be both acceptable and feasible [17]. However, further research is required to better understand the outcomes of the co-design element of ELLY. In particular, evidence on the factors that facilitated community engagement, empowerment and belonging throughout the project life course would inform future commissioning of co-design interventions [7–9]

A small number of participants found the collection of outcome measures burdensome and lengthy. Some outcome measures (in particular, weight measurement and asking about mental health) were found to be intrusive by a few participants, who queried their relevance. Should ELLY be evaluated in a larger scale study, greater attention should be paid to explaining the importance of measures being collected, alongside building in flexibility around how and where measures are collected to align with participants’ preferences.

As ELLY utilised existing assets in communities, the intervention is heavily reliant on what the community offers. In both communities, there was an expectation that the variety, focus and timing of assets would be more wide ranging. Participants expressed that many assets were daytime based and felt female oriented. In addition, some assets were felt to be more welcoming and engaged in ELLY than others. Such limitations reduce the ability to incorporate effective features, such as tailoring, in co-designed community interventions [28].

Some participants felt that communication between assets, volunteers, researchers and ELLY participants was poor. For example, when assets did not run, this was not communicated to the ELLY team and/or participants. A recommendation for a future implementation of the ELLY intervention would be the introduction of a [digital] community board where updates, questions and general information could be posted to keep participants up to date. Although the research team did try to promote the ELLY website and ELLY social media posts as a means of communication, this was not widely used in the study, which may reflect broader aspects of inequality and digital inclusion as well as the (older) age profile of ELLY participants. Structural barriers to digital access may need consideration in future interventions with such groups. [29]

ELLY was implemented across two communities by community volunteers and partner organisations. Different contexts, reflected in aspects such as the existing sense of community, local politics, and pivotal community members, shaped how the intervention was delivered and received. In addition, differences in key mechanisms of action such as community champions of the intervention, level of community and assets’ buy-in to the intervention resulted in very different models of implementation across the communities. Despite these differences in local adaption, findings were broadly comparable across communities. This suggests that the ELLY intervention has the potential to maintain fidelity whilst allowing for flexibility to adapt to varied community contexts, without compromising its core functions or the achievement of similar outcomes for participants.

### Contribution to the literature

Findings from our study are in line with the literature that support weight inclusive (rather than weight normative) approaches, community empowerment, and shifting models of care further upstream [11,12]. Similar findings have been reported in other research indicating that a holistic approach is acceptable and feasible to this population group. This is an important consideration for the ‘framing’ of future interventions and engaging similar population groups [30].

Investing in upstream community incentive initiatives that are feasible and acceptable warrants further investigation to explore their potential to reduce pressure on existing health services [6]. Addressing the value of public health interventions and the return on investment of weight-loss programmes along with the possible negative impact on wellbeing is important. Previous research on weight stigma shows significant negative impact on wellbeing [11,12], highlighting a need to support wellbeing as well as weight-focused goals [30]. The recent focus on human learning systems, where choice and autonomy is given to individuals, show promise in empowering individuals to take control and set their own measurements of what matters to them. [9,31] The ELLY intervention, co-designed by our communities, reflects this approach where community participants wanted autonomy, wanted choice around goals set and wanted a holistic approach to healthy weight and wellbeing rather than a focus on individual, potentially stigmatising conditions like weight or behaviour.

## Conclusions

This study successfully demonstrates the feasibility of the ELLY intervention, with the qualitative component of the work providing insight into how participants perceived ELLY had supported healthy weight and wellbeing. Findings suggest wellbeing and social connection, rather than healthy weight, were more closely associated with positive changes in behaviour, with achieving or maintaining healthy weight potentially reflecting participants’ increased engagement in community assets and social connections, and healthier day-to-day routines. Improvements in self-efficacy and confidence also appeared to act as a catalyst for further positive behaviour change.

There are limitations to the study findings. Although interviewees represented 27% of all participants and included participants with varying levels of engagement in the intervention, the sample did not include participants that dropped out of the study (n = 9) who may have provided important insights that could have challenged or counterbalanced the findings. There is the potential that interviewees felt compelled to give expected answers and interviewer influence cannot be dismissed; however, participants were briefed to be open and honest and experienced researchers took care to avoid biasing responses. Further work is warranted to determine the transferability and relevance of our logic model to other communities and contexts, particularly considering the variability of findings across the communities in this study. In addition, a longer-term study is required to further explore immediate and intermediate outcomes of the ELLY intervention, whether and how it may contribute to sustain behaviour change in the long-term. Moreover, an effectiveness and cost-effectiveness trial is required to assess the potential of such upstream, community co-designed approaches to supporting citizens living in disadvantaged communities to live healthy lives. Notwithstanding these limitations, the richness and depth of our findings provide important insights into participants’ experiences and perceived pathways to outcomes, advancing understanding of how community interventions may support health and wellbeing and informing future research in this area.

## Supporting information

S1 Appendix COREQ 32-item checklist. (DOCX)

S2 Appendix Interview Topic Guide. (DOCX)

S3 Appendix Additional quotes from interviews. (DOCX)

## References

[1] Office of National Statistics. Deaths involving COVID-19 by local area and socioeconomic deprivation: deaths occurring between 1 March and 31 July 2020. Available from: www.ons.gov.uk/peoplepopulationandcommunity/birthsdeathsandmarriages/deaths/bulletins/deathsinvolvingcovid19bylocalareasanddeprivation/deathsoccurringbetween1marchand31july2020#english-index-of-multiple-deprivation

[2] Obesity Action Scotland: Healthy weight for all Factsheet 2023/24 Data. 2025. https://www.obesityactionscotland.org/media/sw5ome13/obesity-in-scotland-prevalence-causes-impact-and-responses-march-2025.pdf

[3] Lee A, Cardel M, Donahoo WT. Social and environmental factors influencing obesity. In: Feingold KR, Ahmed SF, Anawalt B, editors. Endotext. South Dartmouth (MA): MDText.com, Inc. 2000. 25905211

[4] Van Der Velde LA, Schuilenburg LA, Thrivikraman JK, Numans ME, Kiefte-de Jong JC. Needs and perceptions regarding healthy eating among people at risk of food insecurity: a qualitative analysis. Int J Equity Health. 2019;18(1):184. 10.1186/s12939-019-1077-0 PMC6880580 31775770

[5] Watts P, Buck D, Netuveli G, Renton A. Clustering of lifestyle risk behaviours among residents of forty deprived neighbourhoods in London: lessons for targeting public health interventions. J Public Health (Oxf). 2016;38(2):308–15. doi: 10.1093/pubmed/fdv028 25762701 PMC4894479

[6] Brunton G, Thomas J, O’Mara-Eves A, Jamal F, Oliver S, Kavanagh J. Narratives of community engagement: a systematic review-derived conceptual framework for public health interventions. BMC Public Health. 2017;17(1):944. doi: 10.1186/s12889-017-4958-4 29228932 PMC5725895

[7] Langford R, Brockman R, Banks J, Jago R, Gillison F, Coulman K, et al. Co-designing adult weight management services: a qualitative study exploring barriers, facilitators, and considerations for future commissioning. BMC Public Health. 2024;24(1):778. doi: 10.1186/s12889-024-18031-w 38475750 PMC10935989

[8] Peters S, Guccione L, Francis J, Best S, Tavender E, Curran J, et al. Evaluation of research co-design in health: a systematic overview of reviews and development of a framework. Implement Sci. 2024;19(1):63. doi: 10.1186/s13012-024-01394-4 39261956 PMC11391618

[9] Bolland C, Robins A, Gray P, Sindall P. Empowerment in obesity and behavioral weight‐management programmes: a scoping review. Obes Rev. 2026. doi: 10.1111/obr.70141 PMC13655984 42036136

[10] South J, Connolly AM, Stansfield JA, Johnstone P, Henderson G, Fenton KA. Putting the public (back) into public health: leadership, evidence and action. J Public Health (Oxf). 2019;41(1):10–7. doi: 10.1093/pubmed/fdy041 29546426

[11] Talumaa B, Brown A, Batterham RL, Kalea AZ. Effective strategies in ending weight stigma in healthcare. Obes Rev. 2022;23(10):e13494. doi: 10.1111/obr.13494 35934011 PMC9540781

[12] Alimoradi Z, Golboni F, Griffiths MD, Brostrom A, Chung-Ying L, Pakpour AH. Weight-related stigma and psychological distress: A systematic review and meta-analysis. Clin Nutr. 10.1016/j.clnu.2019.10.016 31732288

[13] Sharpe PA, Bell BA, Liese AD, Wilcox S, Stucker J, Hutto BE. Effects of a food hub initiative in a disadvantaged community: A quasi-experimental evaluation. Health Place. 2020;63:102341. doi: 10.1016/j.healthplace.2020.102341 32543428 PMC7357735

[14] Hoddinott P, O’Dolan C, Macaulay L, Dombrowski SU, Swingler J, Cotton S, et al. Text messages with financial incentives for men with obesity: a randomized clinical trial. JAMA. 2024;332(1):31–40. doi: 10.1001/jama.2024.7064 38744430 PMC11094620

[15] Boonmanunt S, Pattanaprateep O, Ongphiphadhanakul B, McKay G, Attia J, Vlaev I. Evaluation of the effectiveness of behavioral economic incentive programs for goal achievement on healthy diet, weight control and physical activity: a systematic review and network meta-analysis. Ann Behav Med. 2023;57(4):277–87. doi: 10.1136/bmjopen-2020-046035 PMC10094952 36367428

[16] Nickel S, von dem Knesebeck O. Effectiveness of community-based health promotion interventions in urban areas: a systematic review. J Community Health. 2020;45(2):419–34. doi: 10.1007/s10900-019-00733-7 31512111

[17] Cowie J, Findlay S, Archibald R, Currie S, Campbell P, Hutcheon D, et al. Feasibility study of a co-designed, evidence-informed and community-based incentive intervention to promote healthy weight and well-being in disadvantaged communities in Scotland. BMJ Open. 2025;15(2):e092908. doi: 10.1136/bmjopen-2024-092908 39979047 PMC11843023

[18] Morgan H, Hoddinott P, Thomson G, Crossland N, Farrar S, Yi D, et al. Benefits of Incentives for Breastfeeding and Smoking cessation in pregnancy (BIBS): a mixed-methods study to inform trial design. Health Technol Assess. 2015;19(30):1–522, vii–viii. doi: 10.3310/hta19300 25897655 PMC4780978

[19] Thomson G, Morgan H, Crossland N, Bauld L, Dykes F, Hoddinott P, et al. Unintended consequences of incentive provision for behaviour change and maintenance around childbirth. PLoS One. 2014;9(10):e111322. doi: 10.1371/journal.pone.0111322 25357121 PMC4214733

[20] Hoffmann TC, Glasziou PP, Boutron I, Milne R, Perera R, Moher D, et al. Better reporting of interventions: template for intervention description and replication (TIDieR) checklist and guide. BMJ. 2014;348:g1687. doi: 10.1136/bmj.g1687 24609605

[21] Lee RM, Draper M, Lee S. Social connectedness, dysfunctional interpersonal behaviors, and psychological distress: testing a mediator model. J Couns Psychol. 2001;48(3):310. doi: 10.1037/0022-0167.48.3.310

[22] Tong A, Sainsbury P, Craig J. Consolidated criteria for reporting qualitative research (COREQ): a 32-item checklist for interviews and focus groups. Int J Qual Health Care. 2007;19(6):349–57. doi: 10.1093/intqhc/mzm042 17872937

[23] Malterud K, Siersma VD, Guassora AD. Sample size in qualitative interview studies: guided by information power. Qual Health Res. 2016;26(13):1753–60. doi: 10.1177/1049732315617444 26613970

[24] Ritchie J, Ormston R, McNaughton Nicholls C, Lewis J. Qualitative research practice: A guide for social science students and researchers. 2nd ed. Sage Publication. 2013.

[25] West R, Michie S. A brief introduction to the COM-B model of behaviour and the PRIME theory of motivation. 10.32388/WW04E6.2

[26] Ogden J, Karim L, Choudry A, Brown K. Understanding successful behaviour change: the role of intentions, attitudes to the target and motivations and the example of diet. Health Ed Res. 2007;22(3):397–405. doi: 10.1093/her/cyl090 16971672

[27] Carrillo-Álvarez E, Kawachi I, Riera-Romaní J. Neighbourhood social capital and obesity: a systematic review of the literature. Obes Rev. 2019;20(1):119–41. doi: 10.1111/obr.12760 30306717

[28] Gkiouleka A, Harasgama S, Pearce H, Johnson L, Kuhn I, Lunn AD, et al. Improving behavioural interventions in healthcare to address the needs of disadvantaged groups: A policy-focused evidence brief. Public Health Pract (Oxf). 2026;11:100780. doi: 10.1016/j.puhip.2026.100780 42273191 PMC13247692

[29] Holmes H, Burgess G. Digital exclusion and poverty in the UK: How structural inequality shapes experiences of getting online. Digital Geography and Society. 2022;3:100041. doi: 10.1016/j.diggeo.2022.100041

[30] Hunger JM, Smith JP, Tomiyama AJ. An evidence‐based rationale for adopting weight‐inclusive health policy. Soc Issues Policy Rev. 2020;14(1):73–107. doi: 10.1111/sipr.12062

[31] Lowe T, French M, Hawkins M, Hesselgreaves H, Wilson R. New development: Responding to complexity in public services—the human learning systems approach. Pub Money Manag. 2020;41(7):573–6. doi: 10.1080/09540962.2020.1832738

