# Supplementary material for: Enjoy Life LocallY (ELLY): A qualitative study of a co-designed healthy weight and wellbeing intervention in two disadvantaged Scottish communities
Source: PLoS One. 2026 Nov 5;21(11):e0361989. doi: 10.1371/journal.pone.0361989 (PMC13739139; doi:10.1371/journal.pone.0361989)
Supplement: Supplementary file 1 [file pone.0361989.s004.pdf]

## PONE-D-26-07224 Review Report

➢ **Title of the Manuscript:**

**Enjoy Life LocallY (ELLY): A qualitative study of participant perspectives of the community co-designed intervention to support healthy weight and wellbeing in two disadvantaged Scottish communities**

➢ **Summary of the Manuscript:**

This manuscript presents the qualitative component of the ELLY feasibility study, a community co-designed, incentive-based intervention implemented in two disadvantaged Scottish communities to support healthy weight and wellbeing. The intervention included free soup provision, engagement with community assets through a loyalty-card incentive mechanism, goal setting, and optional self-monitoring. Semi-structured interviews with 20 participants explored feasibility, acceptability, and perceived impacts. The study reports improvements in social connectedness, behavioural motivation, mental wellbeing, dietary awareness, and community engagement. A conceptual logic model was developed to explain pathways linking intervention components with outcomes.

➢ **Strengths of the Manuscript:**

- The study addresses a highly relevant public health issue involving obesity, wellbeing, and health inequalities among disadvantaged populations.
- Community co-design and participatory implementation are important strengths and improve contextual relevance.
- The intervention is innovative in combining incentives, community engagement, and wellbeing-focused approaches rather than solely concentrating on weight reduction.
- The qualitative approach provides rich participant perspectives and captures social and behavioural dimensions often missed in quantitative studies.
- The manuscript attempts to conceptualise pathways to outcomes through logic models, which adds theoretical value.
- Use of reporting frameworks such as TIDieR and COREQ improves transparency of intervention reporting.
- The study successfully engaged underserved communities that are often difficult to recruit into lifestyle interventions.

➢ **Overall Impact of the Manuscript:**

The manuscript has potential contribution to the literature on community-based healthy weight and wellbeing interventions in disadvantaged populations. The intervention itself appears meaningful and socially relevant. However, the manuscript currently suffers from major weaknesses in scientific presentation, methodological clarity, consistency, reporting quality, language structure, and manuscript organisation. Considerable revision is necessary before the

manuscript can be considered for publication. The discussion and conclusion sections, in particular, require substantial improvement to ensure scientific coherence and interpretive alignment.

- **Section-Wise Evaluation**

**Title:** The title adequately reflects the study focus, intervention, and qualitative design. However, it is somewhat lengthy and could be simplified for readability.

**Abstract:** The abstract provides a broad overview of the intervention and findings but has several important deficiencies:

- The study area is not clearly mentioned.
- The study population is insufficiently described.
- The study period is absent.
- Keywords are missing entirely.
- Important methodological details, particularly regarding qualitative analysis and evaluation of health and wellbeing, are not provided.
- Some sentences are incomplete or grammatically fragmented, especially around lines 29-32.
- The findings remain overly general and descriptive.

The abstract requires substantial restructuring and clarification.

**Keywords:** Keywords are not provided in the manuscript. This is a significant omission. Appropriate indexing terms should be added.

**Introduction:** The introduction establishes the public health relevance of obesity and deprivation reasonably well. However:

1. The geographical context is not consistently clear. At line 36, "Office for National Statistics" is mentioned without specifying the country. Similar ambiguity occurs throughout the Introduction section.
2. The literature review lacks deeper critical synthesis.
3. Some sentences appear incomplete or poorly constructed.
4. The statement on lines 66-67 ("The study was conducted in two phases…") requires clearer wording and better sentence structure.
5. The rationale and research gap should be more explicitly articulated.
6. Abbreviations such as SIMD are introduced inconsistently.

Overall, the Introduction requires language refinement and improved contextual clarity.

**Methods:** The methods section contains useful operational details but lacks important methodological clarity:

1. There is insufficient explanation regarding how “health and wellbeing” were evaluated.
2. Although snowball recruitment is discussed, it is unclear how the first-level participants were initially recruited.
3. The qualitative methodology lacks explicit theoretical or epistemological positioning.
4. Sampling procedures require greater transparency.
5. Data saturation is not discussed.
6. Reflexivity and researcher positionality are inadequately addressed.
7. The process for development of the conceptual logic model is insufficiently explained.
8. It is unclear whether interview coding disagreements occurred and how they were resolved.
9. Abbreviations related to investigators/authors (SF, RA, JC, DH) are not clearly introduced. Their use within the manuscript is somewhat confusing and should be reconsidered or formally clarified.
10. The manuscript inconsistently applies abbreviations such as C1/C2, SIMD, and other short forms.

Methodological rigour and reporting clarity require improvement.

**Ethical approval:** Ethical approval is appropriately reported. However, additional information regarding confidentiality safeguards, management of sensitive disclosures, and participant protections would strengthen ethical transparency.

**Results:** The results section contains meaningful participant narratives and identifies several important themes. However:

1. Thematic organisation lacks coherence in some places.
2. At line 304, “Improved diet” emerges as a new theme, although healthier diet appears conceptually linked to the intervention aims and should have been integrated more systematically earlier in the manuscript.
3. Several participant quotations appear truncated or poorly formatted.
4. Quantification of qualitative responses is inconsistently applied.
5. Some themes remain descriptive and insufficiently analytical.
6. Negative or contradictory experiences are underexplored.
7. The sequence and organisation of participants in Table 1 are unclear and should be justified or standardised.

The results require stronger thematic integration and improved presentation quality.

**Tables & Figures:**

1. Table 1 lacks clarity regarding sequencing and participant ordering.
2. Figures are of insufficient quality and readability. Several diagrams are difficult to interpret.
3. Placeholder statements such as "<insert figure here>" remain within the manuscript and are unacceptable in a submission-ready document.
4. Some figure captions are inaccurate or repetitive.
5. The conceptual diagrams require improved formatting, readability, and explanatory detail.
6. Supplementary files A and B are referred to in the manuscript but are not attached. This is a major presentation issue.

Considerable improvement in figure quality and manuscript preparation is required.

**Discussion:** The Discussion section addresses potentially important implications regarding wellbeing, social connectedness, and behaviour change. However:

1. The writing is frequently clumsy and difficult to follow. This problem is recurrent throughout the Discussion section.
2. The statement on lines 406-410 is particularly unclear and requires complete rewriting.
3. Some interpretations appear repetitive and descriptive rather than analytical.
4. Causal implications are overstated despite the qualitative feasibility design.
5. There is insufficient critical comparison with existing literature.
6. The placeholder citation of reference 10 at line 416 appears unnecessary.
7. At line 419, the placeholder appears more appropriate for reference 10 rather than reference 15.
8. There is poor comparability and continuity between the Discussion and Conclusion sections. This seriously affects the scientific coherence of the manuscript. The relationship between discussion and conclusion is fundamental to manuscript quality, and currently this alignment is weak.

The Discussion section requires major rewriting for clarity, coherence, and scientific interpretation.

**Conclusions:** The conclusions are broadly aligned with the study aims but are not sufficiently integrated with the arguments developed in the Discussion section. The conclusion appears disconnected from several interpretive points raised earlier. Greater coherence between findings, discussion, and conclusion is necessary. Claims regarding broader applicability and effectiveness should also be presented more cautiously.

**Strengths/Limitations:** The manuscript identifies some limitations but important concerns remain insufficiently addressed:

- Small qualitative sample size.
- Potential self-selection bias.
- Social desirability bias.
- Lack of perspectives from non-participants or dropouts.
- Limited transferability.
- Variability between communities.
- Potential researcher influence during interviews.

The limitations section requires deeper critical reflection.

**Conflict of interest:** A clear conflict of interest statement is not visible and should be included explicitly.

**Funding & Acknowledgements:** Funding and acknowledgements are appropriately included. However, the role of funders in study implementation and interpretation should be clarified more explicitly.

**Author's contributions:** An author contribution statement is absent and should be included according to journal guidelines.

**References:** The references are generally relevant but there are multiple issues:

1. Citation formatting is inconsistent.
2. References 16 and 18 are not appropriately cited within the manuscript text.
3. Several references contain spacing and punctuation inconsistencies.
4. Some URLs and DOI formatting require correction.
5. Reference integration within the Discussion section requires improvement.
