# Supplementary material for: Enjoy Life LocallY (ELLY): A qualitative study of a co-designed healthy weight and wellbeing intervention in two disadvantaged Scottish communities
Source: PLoS One. 2026 Nov 5;21(11):e0361989. doi: 10.1371/journal.pone.0361989 (PMC13739139; doi:10.1371/journal.pone.0361989)
Supplement: Supplementary file 2 [file pone.0361989.s001.docx]

**Consolidated criteria for reporting qualitative studies (COREQ): 32-item checklist**

Developed from: Tong A, Sainsbury P, Craig J. Consolidated criteria for reporting qualitative research (COREQ): a 32-item checklist for interviews and focus groups. International Journal for Quality in Health Care. 2007. doi: [10.1093/intqhc/mzm042](https://doi.org/10.1093/intqhc/mzm042)

| **Item No** | | **Guide Questions/Description** | |  |
| --- | --- | --- | --- | --- |
| 1. Interviewer/ facilitator | | SF and RA conducted all the interviews. | |  |
| 2. Credentials | | SF has an MSc. RA has a Professional Doctorate. | |  |
| 3. Occupation | | SF was the project research assistant. RA was NHS Forth Valley Nutrition Lead | |  |
| 4. Gender | | SF is male. RA is female | |  |
| 5. Experience and training | | Both SF and RA have experience conducting interviews from previous projects. RA supported SF with first 5 interviews. | |  |
| 6. Relationship established | | Both interviewers were known to ELLY participants from recruitment and data collection. | |  |
| 7. Participant knowledge of the interviewer | | Participants were informed that SF and RA were independent researchers interested in finding out how interviewees experienced the ELLY intervention. | |  |
| 8. Interviewer characteristics | | Participants were informed that the interviewer was independent and interested in hearing both positive and negative aspects of ELLY. | |  |
|  | 9. Methodological orientation and Theory | Thematic analysis. | |  |
|  | 10. Sampling | Purposive sampling and snowballing were used in recruitment. | |  |
|  | 11. Method of approach | Participants consented to being interviewed at baseline data collection. Those selected for interview were informed at the 12-week data collection. | |  |
|  | 12. Sample size | 20 participants took part in interviews | |  |
|  | 13. Non-participation | All participants asked to participate in interviews agreed to take part. | |  |
|  | 14. Setting of data collection | Community settings or at interviewee’s home (depending on interviewee’s preference) | |  |
|  | 15. Presence of nonparticipants | No | |  |
|  | 16. Description of sample | Demographic data (gender, age, SIMD). These are described in full in Table 1. | |  |
|  |  | |  | |
|  | 17. Interview guide | Yes. Interview topic guides were co-designed with community partners and piloted on two community members. | |  |
|  | 18. Repeat interviews | No | |  |
|  | 19. Audio/visual recording | Audio recording | |  |
|  | 20. Field notes | The audio recording was accompanied by a researcher diary which included observations and impressions that were not recorded, such as the non-verbal communication of the participant. Field notes were used in the analysis of the results afterwards. | |  |
|  | 21. Duration | Semi-structured interviews lasted approximately 30 minutes on average. | |  |
|  | 22. Data saturation | Data saturation was discussed with the research team. | |  |
|  | 23. Transcripts returned | No | |  |
|  | 24. Number of data coders | Coding was performed by two researchers (DH and RA) and reviewed by JC. | |  |
|  | 25. Description of the coding tree | No coding tree was used. The themes were derived from the data. | |  |
|  | 26. Derivation of themes | The themes were derived from the data and were discussed and agreed by all the authors. | |  |
|  | 27. Software | NVivo 12 | |  |
|  | 28. Participant checking | During the interviews, the interviewers repeated and summarised the participants´ answers to ask for clarification and confirmation of the researcher’s interpretation of the answers. At the end of the interview, the researcher summarised the content to ensure the researcher understood the main content correctly. | |  |
|  | 29. Quotations presented | The results section is illustrated with speech acts as quotes from the participants. Each quote is identified with a participant number. | |  |
|  | 30. Data and findings consistent | The data presented in the study and the results that emerge from them are consistent. | |  |
|  | 31. Clarity of major themes | The major themes are presented in the results section of our article. | |  |
|  | 32. Clarity of minor themes | Minor themes arising are discussed within the major themes’ sections of the paper. | |  |
