# Supplementary material for: Enjoy Life LocallY (ELLY): A qualitative study of a co-designed healthy weight and wellbeing intervention in two disadvantaged Scottish communities
Source: PLoS One. 2026 Nov 5;21(11):e0361989. doi: 10.1371/journal.pone.0361989 (PMC13739139; doi:10.1371/journal.pone.0361989)
Supplement: Supplementary file 3 [file pone.0361989.s002.docx]

**ELLY (Enjoy Life LocallY) Project**

**12-week**

**Interview topic guide – Participants**

**1. Introduction**

*Introduce yourself*

*Thank participant for agreeing to chat about their experiences of being involved in the ELLY project.*

*Really value what you have to say, as it will help us design future projects and improve experiences of participants.*

*Information is confidential so anything you say will not be traced back to you. We are asking consent to record our discussion, to help us to accurately remember what you tell us.*

*Please speak about your own views throughout, rather than what other people might think.*

*If you are happy to go ahead, please review and sign the consent form. Are you happy to go ahead with the interview? Great, Let’s get started.*

**Participant, engagement and goal setting:** I’d like to hear your ELLY story. Thinking back over the 12 weeks you’ve been involved in ELLY, tell me about what it’s been like (*Prompts: soup involvement, activities attended).*

Prompts if not mentioned

- How did you hear about the Elly project? Prompt: *Posters, WOM, social media etc.*
- What motivated you to participate? *Prompt: incentives, improve wellbeing, friends/ family, other?*
- What did you expect from being involved in the ELLY project?
- Tell me about how you decided on what goals you might set for the ELLY project.
- Tell me how you found the process of setting goals for yourself.
- How did you think ELLY might help you achieve the goals you set?

**Soup cafes:** Tell me about your experiences of the twice weekly ELLY soup.

Prompts if not mentioned

- How did going along / collecting soup / soup delivery make you feel?
- Thinking about the goals that you set at the start of the project (personal goal, weight goal and wellbeing goal) what are your thoughts on the role of ELLY soup in helping you achieve your goals? (prompt: why do you think this?)
- Are there things that made it easy for you to participate?
- Are there things that would make the soup cafes better?
- Overall, what did you think of this part of the ELLY project?

**Local activities:** Tell me about your experiences of attending local activities during ELLY.

Prompts if not already mentioned

- How do the activities you attended during the ELLY project compare to things that you used to do before you started the project?
- What activities did you enjoy the most?
- Thinking about the goals that you set at the start of the project (personal goal, weight goal and wellbeing goal) what are your thoughts on the role of the activities in helping you achieve your goals? (prompt: why do you think this?)
- Tell me about any unexpected things that you got out of attending the activities, for example, new friends, getting out more, learning new skills?
- Are there things that made it easy for you to participate in activities?
- Are there things that would make the activities better?
- Overall, what did you think of this part of the ELLY project?

**ELLY Loyalty card:** How did you feel about the ELLY Loyalty card and reward system?

Prompts if not mentioned

- How did it impact on what you did each week during the ELLY project?
- How did you feel getting the loyalty card stamped at activities?
- What did you think of being rewarded for attending at least one activity each week?
- How did the reward impact on what you did each week during the ELLY project?
- What are your thoughts on the amount of reward you could receive?
- What are your thoughts on ease of use of the Loyalty card?
- What things about the ELLY loyalty card did you like?
- Are there things that would make the ELLY loyalty card work better?
- Overall, what did you think of this part of the ELLY project?

**Reflecting on ELLY project and future plans**

Now you’ve completed the ELLY project, what are your overall thoughts on your experience?

1. If you were telling a neighbour/friend/family member about ELLY, what would you say to them?
2. Would you take part in ELLY again? (*prompt: explain why you gave the answer you did*)

*Future of ELLY*

1. What parts of the project do you think are workable in the long term?
2. Are there any factors that might make Elly soup and support difficult to keep going over time?
3. Have you any thoughts on ways in which ELLY might be funded in the future?
4. What are your thoughts on how much people might be willing to volunteer to support the ELLY project (*prompt: help out in soup café, put on activities*)
5. Based on your experiences, which aspects of the work would you like to see continuing/not continuing in future

Lastly, is there anything else we’ve not touched on that you’d like to share about your experience of the ELLY project?

*(close interview, and thank participant for their time)*
