# Supplementary material for: Enjoy Life LocallY (ELLY): A qualitative study of a co-designed healthy weight and wellbeing intervention in two disadvantaged Scottish communities
Source: PLoS One. 2026 Nov 5;21(11):e0361989. doi: 10.1371/journal.pone.0361989 (PMC13739139; doi:10.1371/journal.pone.0361989)
Supplement: Supplementary file 4 [file pone.0361989.s003.docx]

**Additional quotes to support themes**

Theme: Snowball recruitment

*“I’ve told some of my friends about it and so we’ll probably access it together at some point in time...I have been speaking obviously to people at school about it and I’ve suggested that we go and do some things” [C1, D19]*

*“One girl in particular was saying that she hadn’t eaten for a few days, so I was telling her to go to the Hope Hub because you’ll get a soup on this day and this day....It’s been a really good way for people to get to know about things and to pass that information around as well.” [C1, P26]*

*“I’ve mentioned it to girls at my work because they kind of covered this area...I let them know and they can kind of let patients know that there’s kind of X, Y, and Z, on in the area” [C1, D24]*

Theme: Connecting with community assets

*“It’s been really enjoyable because I didn’t realise half of the things that go on in the area, so it’s been really nice to see what other things that is available for people” [C1, P26]*

*“I think before ELLY I’d gone to the [community voluntary organisation] maybe twice in a year, whereas with the ELLY project I was going probably most weeks” [C1, P26]*

*“I now take my kids out. There they were very more indoor. I told you I was able to take them up to the [local attraction] at night just to see it glow, and they would say, “Wow.” So, this was great! All these evenings that I’d left them with my wife at home.” [C2, P3]*

Theme: Positive changes in behaviour

“*I was going to try and stop smoking…I’m virtually getting to the stage where I’m not smoking at all…So I’ll just keep plodding on until I’m totally smoke free” [C1, P31]*

*“I feel like I have and I feel like I’ve been more healthier in general…. I think maybe meeting new people and trying out new things, which I definitely did” [C1, P26]*

*“I’ve finished the goal…I was crocheting something, so I finished… It was a headache, an absolute headache. But I had to make that goal to complete it. It made me feel better.” [C2, P101]*

*“It breaks up your day. You’re not in the house 24/7 and it’s going to be good for your wellbeing because it gets out of the house and that’s the important thing. Getting out and getting motivated” [C1, P43]*

Theme: Social connectedness

*“it’s an opportunity to meet people…by coming out and being part of it and even like I said, just popping in and seeing who is in the groups, even if I’m not going to stay, it still gives me a chance to talk to them and just communicate and have that connection with them” [C2, P41]*

*“It was more like everybody became friends because everybody was so chatty and just talking away and friendly. So it was like you’d known them for ages. You hadn’t just met them” [C2, P34]*

*“Yeah, it’s getting to know the community and the community gets to know you. Less hatred that way in the community and stuff, everyone gets to know each other, brings people closer” [C1, P30]*

*“It’s a good idea and it’s for the good of the community. Trying to get the community together because let’s face it, after COVID, everybody’s just kind of [apprehensive]” [C2, P19]*

*“Despite whatever’s going on, everyone’s friendly…It’s not just the coffee morning and everything, it’s if you want any help or anything or advice, not just them, even the people there that come to it” [C1, P30]*

*“There’s always someone there…if I could call one of them up right now and start speaking to them, so ask how are they getting on or they’ll call me and ask how I’m getting on and stuff,	that network is there now…and that does help” [C1, P2]*

Theme: Healthier diet

*“She was telling us how to control our portions and how our mind plays with us about eating and then how to half your plate, and instead of eating too much in one go” [C2, P105]*

*“It’s a major hassle, even thinking about what you fancy, and then thinking about actually cooking it, and nine times out of ten even if I have cooked it, by the time I’ve cooked it I’m looking at it and I’m thinking, “No, don’t fancy it.” And it goes in the bucket. Whereas coming up here, I can just think instantly, “What do you fancy?” and they can make me such and such, and nine times out of ten you will eat it” [C2, P27]*

*“…instead of eating too much in one go…I’m actually eating salad now every day” [C2, P105]*

Theme: Health improvements (mental and physical)

*“…this has actually got me going out. Like I said, even walking round the park, and back. Going to the shops, doddling about, things like that. I’m actually venturing out a bit more than what I normally did” [C1, P36]*

*“It boosts your confidence and your mood, it helps me…before that it was a lot to get out of the house…I was stuck in the house all the time, with my depression and that. I’m out of the house every day. I don’t chat all the time but I’m coming out of my shell a bit” [C1, P31]*

*“Not knowing what was happening, meeting people I didn’t know. Going to place I hadn’t been to. Gradually getting used to civilisation again I suppose…* *it was the project that helped me do it. I don’t think I would have done it myself” [C1, P36]*

*“Now, I’m a lot more open, I’m less negative and I feel as though I am more positive now. Yes, it really, really helps” [C2, P2]*

*“…the banter that goes on in the Men’s Shed and that kind of thing, which I missed when I was working but the Men’s Shed’s giving me the banter, you know, finding the banter again” [C2, P19]*

“*It’s been good to be given the information on what’s on in the area and things, to know the times of where to go because I’ve got anxiety of going to new places and new people, but if I know there’s a set time for things then I can get there for that time or in between” [C1,P30]*

*“I had just lost my mum who I had been caring for for three years and living with her, and I felt really … I still do, but like I say I find this is helping. It’s keeping me busy. It’s keeping me active, because when I’m on my own, it’s just me…* *Aye, it’s helping me break that sadness that I am in. It’s helping me break away from it and just try and forget about it for a while” [C2, P27]*

Theme: New skills and knowledge

*“I’m learning how to do wood turning…* *I’m learning how to fix bikes. We’ve got a bit upstairs, it’s fixing bikes, old bikes, trying to recycle, you know” [C2, P19]*

*“they’ve been bringing their own crafts along and asking me to teach them how to crochet” [C2, P101].*

*“It cost me £400, this Cricut maker, and I don’t know how to work it. One of the women, Marian, at the arts and crafts, we have made a date. I think it is January, after Christmas and that, she is going to come to my house one night and teach me how to use this Cricut machine.” [C2, P27]*
