# Supplementary material for: Enjoy Life LocallY (ELLY): A qualitative study of a co-designed healthy weight and wellbeing intervention in two disadvantaged Scottish communities
Source: PLoS One. 2026 Nov 5;21(11):e0361989. doi: 10.1371/journal.pone.0361989 (PMC13739139; doi:10.1371/journal.pone.0361989)
Supplement: Supplementary file 5 [file pone.0361989.s005.docx]

**Addressing editor’s and reviewers’ comments**

Thank you for the very helpful comments provided by the editor and reviewers. In the table below we have detailed points raised and how these have been addressed.

| **Editor comments** | |
| --- | --- |
|  | |
| Comments | Response |
| 1. Please ensure that your manuscript meets PLOS ONE's style requirements, including those for file naming. The PLOS ONE style templates can be found at <https://journals.plos.org/plosone/s/file?id=wjVg/PLOSOne_formatting_sample_main_body.pdf> and  <https://journals.plos.org/plosone/s/file?id=ba62/PLOSOne_formatting_sample_title_authors_affiliations.pdf> | The manuscript now meets PLOS ONE’s style requirements. |
| 2. Please provide additional details regarding participant consent. In the ethics statement in the Methods and online submission information, please ensure that you have specified (1) whether consent was informed and (2) what type you obtained (for instance, written or verbal, and if verbal, how it was documented and witnessed). If your study included minors, state whether you obtained consent from parents or guardians. If the need for consent was waived by the ethics committee, please include this information.  If you are reporting a retrospective study of medical records or archived samples, please ensure that you have discussed whether all data were fully anonymized before you accessed them and/or whether the IRB or ethics committee waived the requirement for informed consent. If patients provided informed written consent to have data from their medical records used in research, please include this information. | Ethics information has now been updated in the online submission information and manuscript, stating that written, informed consent was obtained and that the study consisted of only adults (lines 149-152). |
| 3. Thank you for stating the following in the Acknowledgments Section of your manuscript:  “Funding for the soup cafés was kindly provided by two local businesses (INEOS Group and Syngenta) located in one of our communities. Funding for the ELLY project was supported by the Chief Scientist Office (grant number: HIPS/21/32).”  We note that you have provided funding information that is not currently declared in your Funding Statement. However, funding information should not appear in the Acknowledgments section or other areas of your manuscript. We will only publish funding information present in the Funding Statement section of the online submission form.  Please remove any funding-related text from the manuscript and let us know how you would like to update your Funding Statement. Currently, your Funding Statement reads as follows:  “Funding for the ELLY cafés was kindly provided by two local businesses (INEOS Group and Syngenta) located in one of our communities. Funding for the ELLY project was supported by the Chief Scientist Office (grant number: HIPS/21/32).”  Please include your amended statements within your cover letter; we will change the online submission form on your behalf. | We have removed the funding information from our manuscript as requested.  The funding statement we would like is provided below, and in the resubmission cover letter, as requested.  JC received funding for the ELLY project was supported by the Chief Scientist Office, Scottish Government, grant no: HIPS/21/32 (https://www.cso.scot.nhs.uk/.  JC received funding from the INEOS Group Communities Fund (https://www.ineos.com/charities/community/local-charitable-giving/)  Funders played no role in study design, data collection and analysis, decision to publish, or preparation of the manuscript. |
| 4. We note that the grant information you provided in the ‘Funding Information’ and ‘Financial Disclosure’ sections do not match.  When you resubmit, please ensure that you provide the correct grant numbers for the awards you received for your study in the ‘Funding Information’ section. | This has now been rectified and the correct information added in response to point 3. |
| 5. Thank you for stating the following financial disclosure:  “Funding for the ELLY cafés was kindly provided by two local businesses (INEOS Group and Syngenta) located in one of our communities. Funding for the ELLY project was supported by the Chief Scientist Office (grant number: HIPS/21/32).”  Please state what role the funders took in the study.  If the funders had no role, please state: "The funders had no role in study design, data collection and analysis, decision to publish, or preparation of the manuscript."  If this statement is not correct you must amend it as needed.  Please include this amended Role of Funder statement in your cover letter; we will change the online submission form on your behalf. | This information has now been provided in response to point 3. |
| 6. In the online submission form, you indicated that your data is available only on request from a third party. Please note that your Data Availability Statement is currently missing contact details for the third party, such as an email address or a link to where data requests can be made. Please update your statement with the missing information. | In the online submission form, the statement has now been updated with the missing information and now reads:  All data produced in the present study are available upon reasonable request. Glasgow Caledonian University holds the copyright for the full interview transcripts and may grant data sharing permission on request. Requests should be made to Julie.cowie@gcu.ac.uk or HLSEthicsNursing@gcu.ac.uk  In accordance with GCU security policies and in compliance with GDPR, data are stored on password protected GCU drives for a period of 5 years. After this, the data will be destroyed. |
| 7. PLOS requires an ORCID iD for the corresponding author in Editorial Manager on papers submitted after December 6th, 2016. Please ensure that you have an ORCID iD and that it is validated in Editorial Manager. To do this, go to ‘Update my Information’ (in the upper left-hand corner of the main menu), and click on the Fetch/Validate link next to the ORCID field. This will take you to the ORCID site and allow you to create a new iD or authenticate a pre-existing/ iD in Editorial Manager. | ORCID ID has now been updated. |
| 8. Your ethics statement should only appear in the Methods section of your manuscript. If your ethics statement is written in any section besides the Methods, please move it to the Methods section and delete it from any other section. Please ensure that your ethics statement is included in your manuscript, as the ethics statement entered into the online submission form will not be published alongside your manuscript. | The ethics statement has now been included in the Methods section of the manuscript (lines 149-152). |
| 9. If the reviewer comments include a recommendation to cite specific previously published works, please review and evaluate these publications to determine whether they are relevant and should be cited. There is no requirement to cite these works unless the editor has indicated otherwise. | Thank you. We have reviewed the recommendations to cite published works and incorporated these into the manuscript when felt appropriate. |
| **Reviewer 1 comments to the author** | |
| Comments | Response |
| Reviewer #1: The manuscript addresses an important public health issue and presents a potentially valuable community-based intervention for disadvantaged populations. The study has notable strengths, particularly the community co-designed approach and focus on wellbeing and social connectedness. However, the manuscript requires major revision due to substantial issues with methodological clarity, presentation, reporting consistency, figure quality, language structure, and the coherence between the Discussion and Conclusion sections. The abstract and methods sections are missing important details; abbreviations are used inconsistently; the supplementary files are absent; and several formatting and referencing issues require correction. Detailed section-wise comments and specific recommendations are in the attached review report. | We are very grateful to the reviewer for their detailed and extremely helpful comments. We have sought to address these and believe the standard of the manuscript is now greatly improved. Specific points from the reviewer’s report have been addressed below. |
| **Reviewer 1 report comments** |  |
| Comments | Response |
| Title: The title adequately reflects the study focus, intervention, and qualitative design. However, it  is somewhat lengthy and could be simplified for readability. | The title has been shortened to aid readability to: “Enjoy Life LocallY (ELLY): A qualitative study of a co-designed healthy weight and wellbeing intervention in two disadvantaged Scottish communities” |
| Abstract: The abstract provides a broad overview of the intervention and findings but has several  important deficiencies:  o The study area is not clearly mentioned.  o The study population is insufficiently described.  o The study period is absent.  o Keywords are missing entirely.  o Important methodological details, particularly regarding qualitative analysis and  evaluation of health and wellbeing, are not provided.  o Some sentences are incomplete or grammatically fragmented, especially around lines 29-  32.  o The findings remain overly general and descriptive.  The abstract requires substantial restructuring and clarification | We have added the following information:   - Area: Forth Valley, Scotland - Population: residents of disadvantaged communities. Total 75. - Study period: September – November 2023. - For clarity, we have also added that interviewees were spread across both communities. - Keywords are not required by the journal - Information on methods used for analysis of data included. - Sentence structure and grammar checked - Findings strengthened - Overall structure of the abstract has been revised to provide greater clarity |
| Keywords: Keywords are not provided in the manuscript. This is a significant omission.  Appropriate indexing terms should be added. | Keywords are not required for PLOS One submissions |
| Introduction: The introduction establishes the public health relevance of obesity and deprivation  reasonably well. However:  1. The geographical context is not consistently clear. At line 36, “Office for National Statistics”  is mentioned without specifying the country. Similar ambiguity occurs throughout the  Introduction section.  2. The literature review lacks deeper critical synthesis.  3. Some sentences appear incomplete or poorly constructed.  4. The statement on lines 66-67 (“The study was conducted in two phases…”) requires clearer  wording and better sentence structure.  5. The rationale and research gap should be more explicitly articulated.  6. Abbreviations such as SIMD are introduced inconsistently.  Overall, the Introduction requires language refinement and improved contextual clarity. | 1. Clarification that it is the U.K Office of National Statistics has been added (line 40). Throughout the introduction, clarification of the location has been added (line 86; lines 100-101; 126) 2. The literature review has been expanded to provide more detail on the topics relevant to this project. 3. Sentences have been reviewed. 4. The statement on lines 66-67 has be reworded to provide clarity (now lines 87-90). 5. The gap in research has been articulated more clearly. 6. SIMD is not used in the introduction. We have checked all abbreviations here, and throughout the document to ensure consistency (lines 126; 129; 130; 136; 220; Table 1). 7. Overall, the introduction has been reworded and restructured throughout which we believe now provides a better clarity of language and context. |
| Methods: The methods section contains useful operational details but lacks important  methodological clarity:  1. There is insufficient explanation regarding how “health and wellbeing” were evaluated.  2. Although snowball recruitment is discussed, it is unclear how the first-level participants  were initially recruited.  3. The qualitative methodology lacks explicit theoretical or epistemological positioning.  4. Sampling procedures require greater transparency.  5. Data saturation is not discussed.  6. Reflexivity and researcher positionality are inadequately addressed.  7. The process for development of the conceptual logic model is insufficiently explained.  8. It is unclear whether interview coding disagreements occurred and how they were  resolved.  9. Abbreviations related to investigators/authors (SF, RA, JC, DH) are not clearly introduced.  Their use within the manuscript is somewhat confusing and should be reconsidered or  formally clarified.  10. The manuscript inconsistently applies abbreviations such as C1/C2, SIMD, and other short  forms.  11. Methodological rigour and reporting clarity require improvement. | 1. Health and wellbeing were analysed using EQ-5D-5L and WEMWBS validated questionnaires (this information has now been added to the manuscript (line 144-148) and via qualitative interviews where participants explored in the interview any changes to their health and wellbeing they had perceived as a result of being involved in ELLY (added on lines 158-160). 2. Additional information about recruitment has been added (lines 154-156). 3. Clarity around the positioning of the methodological approach used is provided in lines 152-154. 4. More detail around sampling conducted is provided in lines 158-159. 5. Discussion of the predefined sample size and data saturation is now provided in lines 165-168. 6. Reflexivity and positionality are now addressed in lines 188-191. 7. The description of the process of constructing the logic model has been rewritten to provide better clarity (lines 205-212). 8. Lines 199-200 provide a statement on how disagreements were resolved. 9. Author initials have now been added to the statement regarding author contributions such that they are formally clarified (lines 610-616) 10. The manuscript has been thoroughly checked and any inconsistency in use of abbreviations addressed. 11. The Methods section has been thoroughly revised to address the points raised and ensure greater clarity of reporting and methodological rigour employed. |
| Ethical approval: Ethical approval is appropriately reported. However, additional information  regarding confidentiality safeguards, management of sensitive disclosures, and participant  protections would strengthen ethical transparency. | Further detail around these topics is now provided in lines 149-152; 176-181. |
| Results: The results section contains meaningful participant narratives and identifies several  important themes. However:  1. Thematic organisation lacks coherence in some places.  2. At line 304, “Improved diet” emerges as a new theme, although healthier diet appears  conceptually linked to the intervention aims and should have been integrated more  systematically earlier in the manuscript.  3. Several participant quotations appear truncated or poorly formatted.  4. Quantification of qualitative responses is inconsistently applied.  5. Some themes remain descriptive and insufficiently analytical.  6. Negative or contradictory experiences are underexplored.  7. The sequence and organisation of participants in Table 1 are unclear and should be justified  or standardised.  8. The results require stronger thematic integration and improved presentation quality | 1. A substantial review of the organisation of themes has been conducted to ensure flow and coherence. 2. Apologies, this should read healthier diet. We have also ensured this is integrated throughout the manuscript. 3. We have reviewed all quotes and ensured all are now correctly formatted and not cut short. 4. We have reviewed and revised the results section to ensure a consistent approach is used in qualifying proportion of interviewees a statement represents. 5. We have reviewed each theme and ensured findings are now explored in greater detail and where appropriate, supported by interview data. 6. Further comment has been added around negative and contradictory experiences reported. 7. Clarification that Table 1 data are ordered by column 1, community number followed by participant number has been added (line 227-228). 8. Thank you for your helpful comment in this regard. We have revised the section substantially to improve integration of results, and rigour of reporting. We believe the section is much stronger as a result. |
| Tables & Figures:  1. Table 1 lacks clarity regarding sequencing and participant ordering.  2. Figures are of insufficient quality and readability. Several diagrams are difficult to interpret.  3. Placeholder statements such as “<insert figure here>” remain within the manuscript and are  unacceptable in a submission-ready document.  4. Some figure captions are inaccurate or repetitive.  5. The conceptual diagrams require improved formatting, readability, and explanatory detail.  6. Supplementary files A and B are referred to in the manuscript but are not attached. 7. This is a major presentation issue.  Considerable improvement in figure quality and manuscript preparation is required. | 1. This has been addressed (see point 7 above) 2. All figures have now been reviewed revised and are now of high readability and quality. 3. Placeholder statements have now been removed. 4. Figure captions have been reviewed and are now accurate and adequately describe what is depicted in each figure. 5. The conceptual diagrams have been reformatted and are now readable and of high quality. The diagrams adequately reflect the pathways to outcomes arising from the data. 6. We apologise for this oversight and thank the reviewer for highlighting this. The files have now been included and labelled in line with PLOS One requirements. Tables and figures presented are now clear, accurate and of high quality. We thank the reviewer for highlighting these issues. |
| Discussion: The Discussion section addresses potentially important implications regarding  wellbeing, social connectedness, and behaviour change. However:  1. The writing is frequently clumsy and difficult to follow. This problem is recurrent  throughout the Discussion section.  2. The statement on lines 406-410 is particularly unclear and requires complete rewriting.  3. Some interpretations appear repetitive and descriptive rather than analytical.  4. Causal implications are overstated despite the qualitative feasibility design.  5. There is insufficient critical comparison with existing literature.  6. The placeholder citation of reference 10 at line 416 appears unnecessary.  7. At line 419, the placeholder appears more appropriate for reference 10 rather than  reference 15.  8. There is poor comparability and continuity between the Discussion and Conclusion sections.  This seriously affects the scientific coherence of the manuscript. The relationship between  discussion and conclusion is fundamental to manuscript quality, and currently this  alignment is weak.  The Discussion section requires major rewriting for clarity, coherence, and scientific interpretation. | 1. Thank you for this helpful comment. The discussion section has been significantly revised, and we believe is now written in a clear and informative manner.  2. Lines 406-410 have now been re-written to improve clarity (now lines 485 -490).  3. We have ensured findings are sufficiently analysed.  4. We have clarified at the start of this section that results are not definitive causal pathways and should be interpreted as such (see lines 487-490)  5. We have extended the literature to ensure we critically reflect on our findings in relation to the existing evidence base  6. The placeholder citation of reference 10 points readers to the article describing the ELLY study mixed-methods findings. As such we feel it important to keep this citation.  7. Thank you for highlighting this error in citation. We have now cited the correct paper, which was not originally included in our reference list (see line 505 ref 27)  8. We have reviewed the discussion and conclusion extensively as we take on board the reviewer’s comments regarding clumsy writing and lack of flow. We now believe the two sections are coherent and complement each other adequately. |
| Conclusions: The conclusions are broadly aligned with the study aims but are not sufficiently  integrated with the arguments developed in the Discussion section. The conclusion appears  disconnected from several interpretive points raised earlier. Greater coherence between findings,  discussion, and conclusion is necessary. Claims regarding broader applicability and effectiveness  should also be presented more cautiously.  Strengths/Limitations: The manuscript identifies some limitations but important concerns  remain insufficiently addressed:  o Small qualitative sample size.  o Potential self-selection bias.  o Social desirability bias.  o Lack of perspectives from non-participants or dropouts.  o Limited transferability.  o Variability between communities.  o Potential researcher influence during interviews.  The limitations section requires deeper critical reflection. | The conclusion section has been reworded and additional paragraphs added to improve cohesion with the discussion section and overall findings of the study.  The strengths and limitations section of the manuscript has been strengthened to address some of the points raised by the reviewer. Namely: sample size, no data from dropouts, variability between communities, social desirability bias, researcher influence, transferability and variability between communities.  Self-selection bias was not at play as the research team chose a diverse sample to interview rather than individuals selecting themselves. |
| Conflict of interest: A clear conflict of interest statement is not visible and should be included  explicitly. | The conflict-of-interest statement is included in the online submission information and the journal states this should not be included in the main manuscript. |
| Funding & Acknowledgements: Funding and acknowledgements are appropriately included.  However, the role of funders in study implementation and interpretation should be clarified more  explicitly. | The role of funders in the study has now been stated explicitly in the online submission form. |
| Author’s contributions: An author contribution statement is absent and should be included  according to journal guidelines. | Apologies for this omission and thank you for highlighting. We have now included an authors’ contribution statement (lines 609-616). |
| References: The references are generally relevant but there are multiple issues:  1. Citation formatting is inconsistent.  2. References 16 and 18 are not appropriately cited within the manuscript text.  3. Several references contain spacing and punctuation inconsistencies.  4. Some URLs and DOI formatting require correction.  5. Reference integration within the Discussion section requires improvement. | We have reviewed references to ensure consistency of reporting, appropriate punctuation, and URLs and DOIs have appropriate links. In addition, the Discussion section has been reviewed to ensure appropriate citations throughout. |
| **Reviewer 2 comments** | |
| Comments | Response |
|  | We really appreciate the helpful feedback and comments provided by the reviewer and have sought to address these individually, as detailed below. We believe the standard of the manuscript is now much improved. |
| 1. Some important papers need to be reviewed and discussed, such as, Bolland, C., Robins, A., Gray, P., & Sindall, P. (2026). Empowerment in Obesity and Behavioral Weight‐Management Programmes: A Scoping Review. Obesity Reviews, e70141. Peters, S., Guccione, L., Francis, J., Best, S., Tavender, E., Curran, J., ... & Klaic, M. (2024). Evaluation of research co-design in health: a systematic overview of reviews and development of a framework. Implementation Science, 19(1), 63. Langford, R., Brockman, R., Banks, J. et al. Co-designing adult weight management services: a qualitative study exploring barriers, facilitators, and considerations for future commissioning. BMC Public Health 24, 778 (2024). <https://doi.org/10.1186/s12889-024-18031-w> | We are grateful to the reviewer in directing us to these articles and have sought to include further insight into the co-design element of the intervention. We have also provided a link to our mixed-methods paper which provides more detail around the study’s feasibility, co-design elements, acceptability and engagement (reference 17). Discussion of our work and that of the articles suggested is found at lines 54-57 (Introduction section) and lines 526 -536 (Discussion & Contribution to the literature Sections). |
| 2. The latest prevalence of obesity or overweight can be provided in the introduction to help the readers get better understanding of the research topic. | We agree this is a helpful addition and have included statistics on data published in March 2025 (lines 42-43) |
| 3. How did you determine the sample size? | Sample size was a pragmatic decision but was also mindful of appropriate sampling in feasibility studies and likelihood of saturation, given 20 interviews. We have added information into the manuscript and cited an appropriate reference on the topic ([23] (lines 165-168)). |
| 4. The current findings can be compared to the qualitative studies using the similar co-designed interventions to better put the current literature into the previous related ones. | Comparison with qualitative studies using a similar co-design approach has now been incorporated. |
