# Supplementary material for: Enjoy Life LocallY (ELLY): A qualitative study of a co-designed healthy weight and wellbeing intervention in two disadvantaged Scottish communities
Source: PLoS One. 2026 Nov 5;21(11):e0361989. doi: 10.1371/journal.pone.0361989 (PMC13739139; doi:10.1371/journal.pone.0361989)
Supplement: Supplementary file 6 [file pone.0361989.s006.docx]

**Reviewer 1: MINOR REVISION**

Thank you for the very helpful comments provided by Reviewer 1. In the table below we have detailed points raised and how these have been addressed.

| **Reviewer 1 report comments** |  |
| --- | --- |
| Comments | Response |
| 1. Please ensure consistency in the interpretation of qualitative findings and avoid causal language. | Thank you for highlighting these inconsistencies and causal language. We have now ensured language used is more appropriate and does not overstate relationships between what was reported and outcomes observed. Specifically:  Abstract  Line 35-36 we have replaced causal language with “support the hypothesis” and “associated with a range of important health outcomes”  Discussion  Line 494 “perceived causal pathways” added.  Line 500 “not perceived by participants as a motivating factor for changing behaviour” replaces *the primary driver of behaviour change for participants within this population group*.  Lines 503-4 “felt by participants” replaces *reported by participants*.  Lines 507-10 “felt by participants suggested” added; *main driver* replaced with “reason for taking part”; *weight as a secondary outcome* replaced with “potentially may reduce weight through”  Line 515 replaced *ELLY can* with “ELLY may”.  Lines 518-19 replaced *achieved from being involved* with “which participants felt was as a result of taking part in ELLY”.  Lines 534-35 replaced *Our study showed* with “Participants in our study reported”.  Lines 527-576 small amendments to language to avoid causal terminology.  Conclusions  Lines 596-600 replaced *Findings show* with “Findings suggest”; replaced *main drivers* with “associated with”; replaced *secondary consequence* with “potentially reflecting”. |
| 2. Please revise the wording concerning “health improvements” in the Abstract | The wording in the Abstract has been revised to “participant-reported health improvements”, as suggested (Line 33). |
| 3. Please reconsider the wording of the Conclusion regarding healthy weight. | Please see above changes to the Conclusion under Comment 1 above. In addition, wording has been amended from *potential pathway* to “how participants perceived ELLY had supported healthy weight and wellbeing” (line 597-8). |
| 4. Please clarify the conceptual positioning of “snowball recruitment” | We thank the reviewer for raising this important point about terminology. On reflection, we agree that snowball recruitment, and 'connecting with community assets' are better conceptualised as mechanisms through which the intervention operated, rather than as outcomes. The manuscript has been updated accordingly. Specifically:  Line 237 Table 2 has been updated to reflect these components as “mechanisms”.  Lines 240-41 replaced *outcome* with “intervention mechanism”.  Lines 256-60 Clarifies that the component is not a standalone outcome but rather a “reach and dissemination” pathway.  Line 259 Descriptor of Fig 2 updated to “Snowball recruitment as a reach and dissemination mechanism”.  Lines 284-86 Clarified component as a mechanism.  Line 287 Descriptor of Fig 3 updated to “Connecting with community assets awareness and engagement mechanism”.  Conclusion  Lines 485-87 Added “The model distinguishes intervention mechanisms, such as snowball recruitment and community connections, from the outcomes they contributed to, such as social connectedness and positive behaviour change.” |
| 5. Please check the presentation of all figures carefully | The figures now all align with the PLOS One figure requirements which has improved visibility of all diagrams. More detail about the relationship between the figures and the conceptual model has been added. |
| 6. Please undertake a final English-language edit. | A final edit has been completed to improve readability and presentation of the manuscript. |
| 7. Please review terminology relating to outcomes. | The manuscript has been reviewed to clearly distinguish between participant reported and objectively measured outcomes. |
| 8. Please be cautious when describing the transferability of the findings. | We have amended the language used in the Conclusions section of this paper accordingly, so as not to overstate the generalisability of our findings.  Specifically:  Line 610 Replaced *generalisability* with “relevance” which feels more appropriate.  Line 612-13 added “whether and how it may contribute”  Lines 612-19 changed the language to reflect the study providing “important insights”, “advancing understanding of how community interventions may support health and wellbeing and informing future research in the area”. |
| 9. Please further clarify the possibility of positive-selection bias among interview participants. | As described in Lines 162-66 in Section ‘Qualitative data collection’, diversity of participants’ engagement in the intervention was achieved through purposive sampling. We have detailed the selection bias (not including those that dropped out of the study) with greater clarity in lines 603-06: “Although interviewees represented 27% of all participants and included participants with varying levels of engagement in the intervention, the sample did not include participants that dropped out of the study (n=9) who may have provided important insights that could have challenged or counterbalanced the findings”. |
| 10. Please undertake a final reference-format check. | As per PLOS reference guidelines ([Submission Guidelines \| PLOS One](https://journals.plos.org/plosone/s/submission-guidelines#loc-references)) the “Available from” and URLs have been removed from published articles leaving only the full DOI. |
