# Supplementary material for: Enjoy Life LocallY (ELLY): A qualitative study of a co-designed healthy weight and wellbeing intervention in two disadvantaged Scottish communities
Source: PLoS One. 2026 Nov 5;21(11):e0361989. doi: 10.1371/journal.pone.0361989 (PMC13739139; doi:10.1371/journal.pone.0361989)
Supplement: Peer review history

# Peer review: Decision Letter 0

**DOI:** 10.1371/journal.pone.0361989.r001

**Authors:** Rachel Suet Kay Chan

10 May 2026

Dear Dr. Cowie,

Please submit your revised manuscript by Jun 24 2026 11:59PM. If you will need more time than this to complete your revisions, please reply to this message or contact the journal office at plosone@plos.org. When you're ready to submit your revision, log on to https://www.editorialmanager.com/pone/ and select the 'Submissions Needing Revision' folder to locate your manuscript file.

- A letter that responds to each point raised by the academic editor and reviewer(s). You should upload this letter as a separate file labeled 'Response to Reviewers'.
- A marked-up copy of your manuscript that highlights changes made to the original version. You should upload this as a separate file labeled 'Revised Manuscript with Track Changes'.
- An unmarked version of your revised paper without tracked changes. You should upload this as a separate file labeled 'Manuscript'.

If you would like to make changes to your financial disclosure, please include your updated statement in your cover letter. Guidelines for resubmitting your figure files are available below the reviewer comments at the end of this letter.

If applicable, we recommend that you deposit your laboratory protocols in protocols.io to enhance the reproducibility of your results. Protocols.io assigns your protocol its own identifier (DOI) so that it can be cited independently in the future. For instructions see: https://journals.plos.org/plosone/s/submission-guidelines#loc-laboratory-protocols. Additionally, PLOS ONE offers an option for publishing peer-reviewed Lab Protocol articles, which describe protocols hosted on protocols.io. Read more information on sharing protocols at https://plos.org/protocols?utm_medium=editorial-email&utm_source=authorletters&utm_campaign=protocols.

As the corresponding author, your ORCID iD is verified in the submission system and will appear in the published article. PLOS supports the use of ORCID, and we encourage all coauthors to register for an ORCID iD and use it as well. Please encourage your coauthors to verify their ORCID iD within the submission system before final acceptance, as unverified ORCID iDs will not appear in the published article. *Only*  the individual author can complete the verification step; PLOS staff *cannot*  verify ORCID iDs on behalf of authors.

We look forward to receiving your revised manuscript.

Kind regards,

Rachel Suet Kay Chan, Ph.D.

Academic Editor

PLOS One

Journal Requirements:

When submitting your revision, we need you to address these additional requirements.

1. Please ensure that your manuscript meets PLOS ONE's style requirements, including those for file naming. The PLOS ONE style templates can be found at

https://journals.plos.org/plosone/s/file?id=wjVg/PLOSOne_formatting_sample_main_body.pdf and

https://journals.plos.org/plosone/s/file?id=ba62/PLOSOne_formatting_sample_title_authors_affiliations.pdf

2. Please provide additional details regarding participant consent. In the ethics statement in the Methods and online submission information, please ensure that you have specified (1) whether consent was informed and (2) what type you obtained (for instance, written or verbal, and if verbal, how it was documented and witnessed). If your study included minors, state whether you obtained consent from parents or guardians. If the need for consent was waived by the ethics committee, please include this information.

If you are reporting a retrospective study of medical records or archived samples, please ensure that you have discussed whether all data were fully anonymized before you accessed them and/or whether the IRB or ethics committee waived the requirement for informed consent. If patients provided informed written consent to have data from their medical records used in research, please include this information.

3. Thank you for stating the following in the Acknowledgments Section of your manuscript:

“Funding for the soup cafés was kindly provided by two local businesses (INEOS Group and Syngenta) located in one of our communities. Funding for the ELLY project was supported by the Chief Scientist Office (grant number: HIPS/21/32).”

We note that you have provided funding information that is not currently declared in your Funding Statement. However, funding information should not appear in the Acknowledgments section or other areas of your manuscript. We will only publish funding information present in the Funding Statement section of the online submission form.

Please remove any funding-related text from the manuscript and let us know how you would like to update your Funding Statement. Currently, your Funding Statement reads as follows:

“Funding for the ELLY cafés was kindly provided by two local businesses (INEOS Group and Syngenta) located in one of our communities. Funding for the ELLY project was supported by the Chief Scientist Office (grant number: HIPS/21/32).”

Please include your amended statements within your cover letter; we will change the online submission form on your behalf.

4. We note that the grant information you provided in the ‘Funding Information’ and ‘Financial Disclosure’ sections do not match.

When you resubmit, please ensure that you provide the correct grant numbers for the awards you received for your study in the ‘Funding Information’ section.

5. Thank you for stating the following financial disclosure:

“Funding for the ELLY cafés was kindly provided by two local businesses (INEOS Group and Syngenta) located in one of our communities. Funding for the ELLY project was supported by the Chief Scientist Office (grant number: HIPS/21/32).”

Please state what role the funders took in the study.  If the funders had no role, please state: "The funders had no role in study design, data collection and analysis, decision to publish, or preparation of the manuscript."

If this statement is not correct you must amend it as needed.

Please include this amended Role of Funder statement in your cover letter; we will change the online submission form on your behalf.

6. In the online submission form, you indicated that your data is available only on request from a third party. Please note that your Data Availability Statement is currently missing contact details for the third party, such as an email address or a link to where data requests can be made. Please update your statement with the missing information.

7. PLOS requires an ORCID iD for the corresponding author in Editorial Manager on papers submitted after December 6th, 2016. Please ensure that you have an ORCID iD and that it is validated in Editorial Manager. To do this, go to ‘Update my Information’ (in the upper left-hand corner of the main menu), and click on the Fetch/Validate link next to the ORCID field. This will take you to the ORCID site and allow you to create a new iD or authenticate a pre-existing iD in Editorial Manager.

8. Your ethics statement should only appear in the Methods section of your manuscript. If your ethics statement is written in any section besides the Methods, please move it to the Methods section and delete it from any other section. Please ensure that your ethics statement is included in your manuscript, as the ethics statement entered into the online submission form will not be published alongside your manuscript.

9. If the reviewer comments include a recommendation to cite specific previously published works, please review and evaluate these publications to determine whether they are relevant and should be cited. There is no requirement to cite these works unless the editor has indicated otherwise.

Additional Editor Comments:

Dear Author/s

The reviewers have recommended a Major Revision.

Their comments are below.

Best regards

[Note: HTML markup is below. Please do not edit.]

Reviewers' comments:

Reviewer's Responses to Questions

**Comments to the Author**

1. Is the manuscript technically sound, and do the data support the conclusions?

Reviewer #1: Partly

Reviewer #2: Partly

**********

2. Has the statistical analysis been performed appropriately and rigorously?

Reviewer #1: N/A

Reviewer #2: Yes

**********

3. Have the authors made all data underlying the findings in their manuscript fully available?

The PLOS Data policy

Reviewer #1: No

Reviewer #2: Yes

**********

4. Is the manuscript presented in an intelligible fashion and written in standard English?

Reviewer #1: No

Reviewer #2: Yes

**********

Reviewer #1: The manuscript addresses an important public health issue and presents a potentially valuable community-based intervention for disadvantaged populations. The study has notable strengths, particularly the community co-designed approach and focus on wellbeing and social connectedness. However, the manuscript requires major revision due to substantial issues with methodological clarity, presentation, reporting consistency, figure quality, language structure, and the coherence between the Discussion and Conclusion sections. The abstract and methods sections are missing important details; abbreviations are used inconsistently; the supplementary files are absent; and several formatting and referencing issues require correction. Detailed section-wise comments and specific recommendations are in the attached review report.

Reviewer #2: “Enjoy Life LocallY (ELLY): A qualitative study of participant perspectives of the community co-designed intervention to support healthy weight and wellbeing in two disadvantaged Scottish communities”（ PONE-D-26-07224）

The aim of this qualitative component of the ELLY study was to conceptualise proposed pathways by which the intervention supported participants’ healthy weight and wellbeing in two disadvantaged communities. Despite most participants choosing a weight goal, the resultant pathways by which ELLY supported participants’ healthy weight and wellbeing indicate that healthy weight was not the primary driver of behaviour change for participants within this population group. Many participants mentioned isolation and felt prior to participating in the ELLY study, this had a significant impact on their mental health. The wellbeing and social elements of ELLY were reported to be the most motivating elements of the intervention which is supported by the literature around wellbeing being important to the mechanism of action of incentives for disadvantaged groups. Overall, this topic is very interesting and the qualitative evidence provide important insights about the experience of a co-designed intervention to support healthy weight and wellbeing. However, some concerns appeared after reading the whole manuscript.

1. Some important papers need to be reviewed and discussed, such as,

Bolland, C., Robins, A., Gray, P., & Sindall, P. (2026). Empowerment in Obesity and Behavioral Weight‐Management Programmes: A Scoping Review. Obesity Reviews, e70141.

Peters, S., Guccione, L., Francis, J., Best, S., Tavender, E., Curran, J., ... & Klaic, M. (2024). Evaluation of research co-design in health: a systematic overview of reviews and development of a framework. Implementation Science, 19(1), 63.

Langford, R., Brockman, R., Banks, J. et al. Co-designing adult weight management services: a qualitative study exploring barriers, facilitators, and considerations for future commissioning. BMC Public Health 24, 778 (2024). https://doi.org/10.1186/s12889-024-18031-w

2. The latest prevalence of obesity or overweight can be provided in the introduction to help the readers get better understanding of the research topic.

3. How did you determine the sample size?

4. The current findings can be compared to the qualitative studies using the similar co-designed interventions to better put the current literature into the previous related ones.

**********

what does this mean?). If published, this will include your full peer review and any attached files.

If you choose “no”, your identity will remain anonymous but your review may still be made public.

**Do you want your identity to be public for this peer review?**  For information about this choice, including consent withdrawal, please see our Privacy Policy

Reviewer #1: **Yes:**  Dr D Anwar Basha, Scientist C, ICMR-NIN, Hyderabad

Reviewer #2: No

**********

[NOTE: If reviewer comments were submitted as an attachment file, they will be attached to this email and accessible via the submission site. Please log into your account, locate the manuscript record, and check for the action link "View Attachments". If this link does not appear, there are no attachment files.]

To ensure your figures meet our technical requirements, please review our figure guidelines: https://journals.plos.org/plosone/s/figures

You may also use PLOS’s free figure tool, NAAS, to help you prepare publication quality figures: https://journals.plos.org/plosone/s/figures#loc-tools-for-figure-preparation.

NAAS will assess whether your figures meet our technical requirements by comparing each figure against our figure specifications.

Attachment Submitted filename: PONE-D-26-07224_Riview Report.pdf

# Author comment: Author response to Decision Letter 1

**DOI:** 10.1371/journal.pone.0361989.r002

14 Jul 2026

Thank you for the very helpful comments provided by the editor and reviewers. Below we have detailed points raised and how these have been addressed.

EDITOR COMMENTS

****************

1. Please ensure that your manuscript meets PLOS ONE's style requirements, including those for file naming. The PLOS ONE style templates can be found at https://journals.plos.org/plosone/s/file?id=wjVg/PLOSOne_formatting_sample_main_body.pdf and

https://journals.plos.org/plosone/s/file?id=ba62/PLOSOne_formatting_sample_title_authors_affiliations.pdf

The manuscript now meets PLOS ONE’s style requirements.

2. Please provide additional details regarding participant consent. In the ethics statement in the Methods and online submission information, please ensure that you have specified (1) whether consent was informed and (2) what type you obtained (for instance, written or verbal, and if verbal, how it was documented and witnessed). If your study included minors, state whether you obtained consent from parents or guardians. If the need for consent was waived by the ethics committee, please include this information. If you are reporting a retrospective study of medical records or archived samples, please ensure that you have discussed whether all data were fully anonymized before you accessed them and/or whether the IRB or ethics committee waived the requirement for informed consent. If patients provided informed written consent to have data from their medical records used in research, please include this information.

Ethics information has now been updated in the online submission information and manuscript, stating that written, informed consent was obtained and that the study consisted of only adults (lines 149-152).

3. Thank you for stating the following in the Acknowledgments Section of your manuscript:

“Funding for the soup cafés was kindly provided by two local businesses (INEOS Group and Syngenta) located in one of our communities. Funding for the ELLY project was supported by the Chief Scientist Office (grant number: HIPS/21/32).”

We note that you have provided funding information that is not currently declared in your Funding Statement. However, funding information should not appear in the Acknowledgments section or other areas of your manuscript. We will only publish funding information present in the Funding Statement section of the online submission form. Please remove any funding-related text from the manuscript and let us know how you would like to update your Funding Statement. Currently, your Funding Statement reads as follows: “Funding for the ELLY cafés was kindly provided by two local businesses (INEOS Group and Syngenta) located in one of our communities. Funding for the ELLY project was supported by the Chief Scientist Office (grant number: HIPS/21/32).” Please include your amended statements within your cover letter; we will change the online submission form on your behalf.

We have removed the funding information from our manuscript as requested. The funding statement we would like is provided below, and in the resubmission cover letter, as requested.

JC received funding for the ELLY project was supported by the Chief Scientist Office, Scottish Government, grant no: HIPS/21/32 (https://www.cso.scot.nhs.uk/.

JC received funding from the INEOS Group Communities Fund (https://www.ineos.com/charities/community/local-charitable-giving/)

Funders played no role in study design, data collection and analysis, decision to publish, or preparation of the manuscript.

4. We note that the grant information you provided in the ‘Funding Information’ and ‘Financial Disclosure’ sections do not match.

When you resubmit, please ensure that you provide the correct grant numbers for the awards you received for your study in the ‘Funding Information’ section.

This has now been rectified and the correct information added in response to point 3.

5. Thank you for stating the following financial disclosure:

“Funding for the ELLY cafés was kindly provided by two local businesses (INEOS Group and Syngenta) located in one of our communities. Funding for the ELLY project was supported by the Chief Scientist Office (grant number: HIPS/21/32).”

Please state what role the funders took in the study. If the funders had no role, please state: "The funders had no role in study design, data collection and analysis, decision to publish, or preparation of the manuscript."

If this statement is not correct you must amend it as needed.

Please include this amended Role of Funder statement in your cover letter; we will change the online submission form on your behalf.

This information has now been provided in response to point 3.

6. In the online submission form, you indicated that your data is available only on request from a third party. Please note that your Data Availability Statement is currently missing contact details for the third party, such as an email address or a link to where data requests can be made. Please update your statement with the missing information.

In the online submission form, the statement has now been updated with the missing information and now reads:

All data produced in the present study are available upon reasonable request. Glasgow Caledonian University holds the copyright for the full interview transcripts and may grant data sharing permission on request. Requests should be made to Julie.cowie@gcu.ac.uk or HLSEthicsNursing@gcu.ac.uk

In accordance with GCU security policies and in compliance with GDPR, data are stored on password protected GCU drives for a period of 5 years. After this, the data will be destroyed.

7. PLOS requires an ORCID iD for the corresponding author in Editorial Manager on papers submitted after December 6th, 2016. Please ensure that you have an ORCID iD and that it is validated in Editorial Manager. To do this, go to ‘Update my Information’ (in the upper left-hand corner of the main menu), and click on the Fetch/Validate link next to the ORCID field. This will take you to the ORCID site and allow you to create a new iD or authenticate a pre-existing/ iD in Editorial Manager.

ORCID ID has now been updated.

8. Your ethics statement should only appear in the Methods section of your manuscript. If your ethics statement is written in any section besides the Methods, please move it to the Methods section and delete it from any other section. Please ensure that your ethics statement is included in your manuscript, as the ethics statement entered into the online submission form will not be published alongside your manuscript.

The ethics statement has now been included in the Methods section of the manuscript (lines 149-152).

9. If the reviewer comments include a recommendation to cite specific previously published works, please review and evaluate these publications to determine whether they are relevant and should be cited. There is no requirement to cite these works unless the editor has indicated otherwise. Thank you. We have reviewed the recommendations to cite published works and incorporated these into the manuscript when felt appropriate.

REVIEWER 1 COMMENTS

********************

GENERAL COMMENT

Reviewer #1: The manuscript addresses an important public health issue and presents a potentially valuable community-based intervention for disadvantaged populations. The study has notable strengths, particularly the community co-designed approach and focus on wellbeing and social connectedness. However, the manuscript requires major revision due to substantial issues with methodological clarity, presentation, reporting consistency, figure quality, language structure, and the coherence between the Discussion and Conclusion sections. The abstract and methods sections are missing important details; abbreviations are used inconsistently; the supplementary files are absent; and several formatting and referencing issues require correction. Detailed section-wise comments and specific recommendations are in the attached review report.

GENERAL RESPONSE

We are very grateful to the reviewer for their detailed and extremely helpful comments. We have sought to address these and believe the standard of the manuscript is now greatly improved. Specific points from the reviewer’s report have been addressed below.

SECTION 1 COMMENTS

Title: The title adequately reflects the study focus, intervention, and qualitative design. However, it

is somewhat lengthy and could be simplified for readability. The title has been shortened to aid readability to: “Enjoy Life LocallY (ELLY): A qualitative study of a co-designed healthy weight and wellbeing intervention in two disadvantaged Scottish communities”

Abstract: The abstract provides a broad overview of the intervention and findings but has several

important deficiencies:

o The study area is not clearly mentioned.

o The study population is insufficiently described.

o The study period is absent.

o Keywords are missing entirely.

o Important methodological details, particularly regarding qualitative analysis and

evaluation of health and wellbeing, are not provided.

o Some sentences are incomplete or grammatically fragmented, especially around lines 29-

32.

o The findings remain overly general and descriptive.

The abstract requires substantial restructuring and clarification

SECTION 1 RESPONSE

We have added the following information:

• Area: Forth Valley, Scotland

• Population: residents of disadvantaged communities. Total 75.

• Study period: September – November 2023.

• For clarity, we have also added that interviewees were spread across both communities.

• Keywords are not required by the journal

• Information on methods used for analysis of data included.

• Sentence structure and grammar checked

• Findings strengthened

• Overall structure of the abstract has been revised to provide greater clarity

SECTION 2 COMMENTS

Keywords: Keywords are not provided in the manuscript. This is a significant omission.

Appropriate indexing terms should be added.

SECTION 2 RESPONSE

Keywords are not required for PLOS One submissions

SECTION 3 COMMENTS

Introduction: The introduction establishes the public health relevance of obesity and deprivation

reasonably well. However:

1. The geographical context is not consistently clear. At line 36, “Office for National Statistics”

is mentioned without specifying the country. Similar ambiguity occurs throughout the

Introduction section.

2. The literature review lacks deeper critical synthesis.

3. Some sentences appear incomplete or poorly constructed.

4. The statement on lines 66-67 (“The study was conducted in two phases…”) requires clearer

wording and better sentence structure.

5. The rationale and research gap should be more explicitly articulated.

6. Abbreviations such as SIMD are introduced inconsistently.

Overall, the Introduction requires language refinement and improved contextual clarity.

SECTION 3 RESPONSE

1. Clarification that it is the U.K Office of National Statistics has been added (line 40). Throughout the introduction, clarification of the location has been added (line 86; lines 100-101; 126)

2. The literature review has been expanded to provide more detail on the topics relevant to this project.

3. Sentences have been reviewed.

4. The statement on lines 66-67 has be reworded to provide clarity (now lines 87-90).

5. The gap in research has been articulated more clearly.

6. SIMD is not used in the introduction. We have checked all abbreviations here, and throughout the document to ensure consistency (lines 126; 129; 130; 136; 220; Table 1).

7. Overall, the introduction has been reworded and restructured throughout which we believe now provides a better clarity of language and context.

SECTION 4 COMMENTS

Methods: The methods section contains useful operational details but lacks important

methodological clarity:

1. There is insufficient explanation regarding how “health and wellbeing” were evaluated.

2. Although snowball recruitment is discussed, it is unclear how the first-level participants

were initially recruited.

3. The qualitative methodology lacks explicit theoretical or epistemological positioning.

4. Sampling procedures require greater transparency.

5. Data saturation is not discussed.

6. Reflexivity and researcher positionality are inadequately addressed.

7. The process for development of the conceptual logic model is insufficiently explained.

8. It is unclear whether interview coding disagreements occurred and how they were

resolved.

9. Abbreviations related to investigators/authors (SF, RA, JC, DH) are not clearly introduced.

Their use within the manuscript is somewhat confusing and should be reconsidered or

formally clarified.

10. The manuscript inconsistently applies abbreviations such as C1/C2, SIMD, and other short

forms.

11. Methodological rigour and reporting clarity require improvement.

SECTION 4 RESPONSE

1. Health and wellbeing were analysed using EQ-5D-5L and WEMWBS validated questionnaires (this information has now been added to the manuscript (line 144-148) and via qualitative interviews where participants explored in the interview any changes to their health and wellbeing they had perceived as a result of being involved in ELLY (added on lines 158-160).

2. Additional information about recruitment has been added (lines 154-156).

3. Clarity around the positioning of the methodological approach used is provided in lines 152-154.

4. More detail around sampling conducted is provided in lines 158-159.

5. Discussion of the predefined sample size and data saturation is now provided in lines 165-168.

6. Reflexivity and positionality are now addressed in lines 188-191.

7. The description of the process of constructing the logic model has been rewritten to provide better clarity (lines 205-212).

8. Lines 199-200 provide a statement on how disagreements were resolved.

9. Author initials have now been added to the statement regarding author contributions such that they are formally clarified (lines 610-616)

10. The manuscript has been thoroughly checked and any inconsistency in use of abbreviations addressed.

11. The Methods section has been thoroughly revised to address the points raised and ensure greater clarity of reporting and methodological rigour employed.

SECTION 5 COMMENTS

Ethical approval: Ethical approval is appropriately reported. However, additional information

regarding confidentiality safeguards, management of sensitive disclosures, and participant

protections would strengthen ethical transparency.

SECTION 5 RESPONSE

Further detail around these topics is now provided in lines 149-152; 176-181.

SECTION 6 COMMENTS

Results: The results section contains meaningful participant narratives and identifies several

important themes. However:

1. Thematic organisation lacks coherence in some places.

2. At line 304, “Improved diet” emerges as a new theme, although healthier diet appears

conceptually linked to the intervention aims and should have been integrated more

systematically earlier in the manuscript.

3. Several participant quotations appear truncated or poorly formatted.

4. Quantification of qualitative responses is inconsistently applied.

5. Some themes remain descriptive and insufficiently analytical.

6. Negative or contradictory experiences are underexplored.

7. The sequence and organisation of participants in Table 1 are unclear and should be justified

or standardised.

8. The results require stronger thematic integration and improved presentation quality

SECTION 6 RESPONSE

1. A substantial review of the organisation of themes has been conducted to ensure flow and coherence.

2. Apologies, this should read healthier diet. We have also ensured this is integrated throughout the manuscript.

3. We have reviewed all quotes and ensured all are now correctly formatted and not cut short.

4. We have reviewed and revised the results section to ensure a consistent approach is used in qualifying proportion of interviewees a statement represents.

5. We have reviewed each theme and ensured findings are now explored in greater detail and where appropriate, supported by interview data.

6. Further comment has been added around negative and contradictory experiences reported.

7. Clarification that Table 1 data are ordered by column 1, community number followed by participant number has been added (line 227-228).

8. Thank you for your helpful comment in this regard. We have revised the section substantially to improve integration of results, and rigour of reporting. We believe the section is much stronger as a result.

SECTION 7 COMMENTS

Tables & Figures:

1. Table 1 lacks clarity regarding sequencing and participant ordering.

2. Figures are of insufficient quality and readability. Several diagrams are difficult to interpret.

3. Placeholder statements such as “<insert figure here>” remain within the manuscript and are

unacceptable in a submission-ready document.

4. Some figure captions are inaccurate or repetitive.

5. The conceptual diagrams require improved formatting, readability, and explanatory detail.

6. Supplementary files A and B are referred to in the manuscript but are not attached. 7. This is a major presentation issue.

Considerable improvement in figure quality and manuscript preparation is required.

SECTION 7 RESPONSE

1. This has been addressed (see point 7 above)

2. All figures have now been reviewed revised and are now of high readability and quality.

3. Placeholder statements have now been removed.

4. Figure captions have been reviewed and are now accurate and adequately describe what is depicted in each figure.

5. The conceptual diagrams have been reformatted and are now readable and of high quality. The diagrams adequately reflect the pathways to outcomes arising from the data.

6. We apologise for this oversight and thank the reviewer for highlighting this. The files have now been included and labelled in line with PLOS One requirements. Tables and figures presented are now clear, accurate and of high quality. We thank the reviewer for highlighting these issues.

SECTION 8 COMMENTS

Discussion: The Discussion section addresses potentially important implications regarding

wellbeing, social connectedness, and behaviour change. However:

1. The writing is frequently clumsy and difficult to follow. This problem is recurrent

throughout the Discussion section.

2. The statement on lines 406-410 is particularly unclear and requires complete rewriting.

3. Some interpretations appear repetitive and descriptive rather than analytical.

4. Causal implications are overstated despite the qualitative feasibility design.

5. There is insufficient critical comparison with existing literature.

6. The placeholder citation of reference 10 at line 416 appears unnecessary.

7. At line 419, the placeholder appears more appropriate for reference 10 rather than

reference 15.

8. There is poor comparability and continuity between the Discussion and Conclusion sections.

This seriously affects the scientific coherence of the manuscript. The relationship between

discussion and conclusion is fundamental to manuscript quality, and currently this

alignment is weak.

The Discussion section requires major rewriting for clarity, coherence, and scientific interpretation.

SECTION 8 RESPONSE

1. Thank you for this helpful comment. The discussion section has been significantly revised, and we believe is now written in a clear and informative manner.

2. Lines 406-410 have now been re-written to improve clarity (now lines 485 -490).

3. We have ensured findings are sufficiently analysed.

4. We have clarified at the start of this section that results are not definitive causal pathways and should be interpreted as such (see lines 487-490)

5. We have extended the literature to ensure we critically reflect on our findings in relation to the existing evidence base

6. The placeholder citation of reference 10 points readers to the article describing the ELLY study mixed-methods findings. As such we feel it important to keep this citation.

7. Thank you for highlighting this error in citation. We have now cited the correct paper, which was not originally included in our reference list (see line 505 ref 27)

8. We have reviewed the discussion and conclusion extensively as we take on board the reviewer’s comments regarding clumsy writing and lack of flow. We now believe the two sections are coherent and complement each other adequately.

SECTION 9 COMMENTS

Conclusions: The conclusions are broadly aligned with the study aims but are not sufficiently

integrated with the arguments developed in the Discussion section. The conclusion appears

disconnected from several interpretive points raised earlier. Greater coherence between findings,

discussion, and conclusion is necessary. Claims regarding broader applicability and effectiveness

should also be presented more cautiously.

Strengths/Limitations: The manuscript identifies some limitations but important concerns

remain insufficiently addressed:

o Small qualitative sample size.

o Potential self-selection bias.

o Social desirability bias.

o Lack of perspectives from non-participants or dropouts.

o Limited transferability.

o Variability between communities.

o Potential researcher influence during interviews.

The limitations section requires deeper critical reflection.

SECTION 9 RESPONSE

The conclusion section has been reworded and additional paragraphs added to improve cohesion with the discussion section and overall findings of the study.

The strengths and limitations section of the manuscript has been strengthened to address some of the points raised by the reviewer. Namely: sample size, no data from dropouts, variability between communities, social desirability bias, researcher influence, transferability and variability between communities.

Self-selection bias was not at play as the research team chose a diverse sample to interview rather than individuals selecting themselves.

Conflict of interest: A clear conflict of interest statement is not visible and should be included

explicitly. The conflict-of-interest statement is included in the online submission information and the journal states this should not be included in the main manuscript.

Funding & Acknowledgements: Funding and acknowledgements are appropriately included.

However, the role of funders in study implementation and interpretation should be clarified more

explicitly. The role of funders in the study has now been stated explicitly in the online submission form.

Author’s contributions: An author contribution statement is absent and should be included

according to journal guidelines. Apologies for this omission and thank you for highlighting. We have now included an authors’ contribution statement (lines 609-616).

SECTION 10 COMMENTS

References: The references are generally relevant but there are multiple issues:

1. Citation formatting is inconsistent.

2. References 16 and 18 are not appropriately cited within the manuscript text.

3. Several references contain spacing and punctuation inconsistencies.

4. Some URLs and DOI formatting require correction.

5. Reference integration within the Discussion section requires improvement.

SECTION 10 RESPONSE

We have reviewed references to ensure consistency of reporting, appropriate punctuation, and URLs and DOIs have appropriate links. In addition, the Discussion section has been reviewed to ensure appropriate citations throughout.

REVIEWER 2 COMMENTS

********************

GENERAL RESPONSE

We really appreciate the helpful feedback and comments provided by the reviewer and have sought to address these individually, as detailed below. We believe the standard of the manuscript is now much improved.

SECTION 1 COMMENTS

1. Some important papers need to be reviewed and discussed, such as,

Bolland, C., Robins, A., Gray, P., & Sindall, P. (2026). Empowerment in Obesity and Behavioral Weight‐Management Programmes: A Scoping Review. Obesity Reviews, e70141.

Peters, S., Guccione, L., Francis, J., Best, S., Tavender, E., Curran, J., ... & Klaic, M. (2024). Evaluation of research co-design in health: a systematic overview of reviews and development of a framework. Implementation Science, 19(1), 63.

Langford, R., Brockman, R., Banks, J. et al. Co-designing adult weight management services: a qualitative study exploring barriers, facilitators, and considerations for future commissioning. BMC Public Health 24, 778 (2024). https://doi.org/10.1186/s12889-024-18031-w

SECTION 1 RESPONSE

1. We are grateful to the reviewer in directing us to these articles and have sought to include further insight into the co-design element of the intervention. We have also provided a link to our mixed-methods paper which provides more detail around the study’s feasibility, co-design elements, acceptability and engagement (reference 17). Discussion of our work and that of the articles suggested is found at lines 54-57 (Introduction section) and lines 526 -536 (Discussion & Contribution to the literature sections).

SECTION 2 COMMENTS

1. The latest prevalence of obesity or overweight can be provided in the introduction to help the readers get better understanding of the research topic.

2. How did you determine the sample size?

3. The current findings can be compared to the qualitative studies using the similar co-designed interventions to better put the current literature into the previous related ones. Comparison with qualitative studies using a similar co-design approach has now been incorporated.

SECTION 2 RESPONSE

1. We agree this is a helpful addition and have included statistics on data published in March 2025 (lines 42-43)

2. Sample size was a pragmatic decision but was also mindful of appropriate sampling in feasibility studies and likelihood of saturation, given 20 interviews. We have added information into the manuscript and cited an appropriate reference on the topic ([23] (lines 165-168)).

3. Comparison with qualitative studies using a similar co-design approach has now been incorporated.

Attachment Submitted filename: ResponseToReviewers.docx

# Peer review: Decision Letter 1

**DOI:** 10.1371/journal.pone.0361989.r003

**Authors:** Rachel Suet Kay Chan

23 Aug 2026

Dear Dr. Cowie,

Thank you for submitting your manuscript to PLOS One. After careful consideration, we feel that it has merit but does not fully meet PLOS One’s publication criteria as it currently stands. Therefore, we invite you to submit a revised version of the manuscript that addresses the points raised during the review process.

Please submit your revised manuscript by Oct 18 2026 11:59PM. If you will need more time than this to complete your revisions, please reply to this message or contact the journal office at plosone@plos.org. When you're ready to submit your revision, log on to https://www.editorialmanager.com/pone/ and select the 'Submissions Needing Revision' folder to locate your manuscript file.

- A letter that responds to each point raised by the academic editor and reviewer(s). You should upload this letter as a separate file labeled 'Response to Reviewers'.
- A marked-up copy of your manuscript that highlights changes made to the original version. You should upload this as a separate file labeled 'Revised Manuscript with Track Changes'.
- An unmarked version of your revised paper without tracked changes. You should upload this as a separate file labeled 'Manuscript'.

If you would like to make changes to your financial disclosure, please include your updated statement in your cover letter. Guidelines for resubmitting your figure files are available below the reviewer comments at the end of this letter.

If applicable, we recommend that you deposit your laboratory protocols in protocols.io to enhance the reproducibility of your results. Protocols.io assigns your protocol its own identifier (DOI) so that it can be cited independently in the future. For instructions see: https://journals.plos.org/plosone/s/submission-guidelines#loc-laboratory-protocols. Additionally, PLOS One offers an option for publishing peer-reviewed Lab Protocol articles, which describe protocols hosted on protocols.io. Read more information on sharing protocols at https://plos.org/protocols?utm_medium=editorial-email&utm_source=authorletters&utm_campaign=protocols.

As the corresponding author, your ORCID iD is verified in the submission system and will appear in the published article. PLOS supports the use of ORCID, and we encourage all coauthors to register for an ORCID iD and use it as well. Please encourage your coauthors to verify their ORCID iD within the submission system before final acceptance, as unverified ORCID iDs will not appear in the published article. *Only*  the individual author can complete the verification step; PLOS staff *cannot*  verify ORCID iDs on behalf of authors.

We look forward to receiving your revised manuscript.

Kind regards,

Rachel Suet Kay Chan, Ph.D.

Academic Editor

PLOS One

Journal Requirements:

If the reviewer comments include a recommendation to cite specific previously published works, please review and evaluate these publications to determine whether they are relevant and should be cited. There is no requirement to cite these works unless the editor has indicated otherwise.

Please review your reference list to ensure that it is complete and correct. If you have cited papers that have been retracted, please include the rationale for doing so in the manuscript text, or remove these references and replace them with relevant current references. Any changes to the reference list should be mentioned in the rebuttal letter that accompanies your revised manuscript. If you need to cite a retracted article, indicate the article’s retracted status in the References list and also include a citation and full reference for the retraction notice.

Additional Editor Comments:

[Note: HTML markup is below. Please do not edit.]

Reviewers' comments:

**Comments to the Author**

Reviewer #1: (No Response)

Reviewer #2: All comments have been addressed

**********

2. Is the manuscript technically sound, and do the data support the conclusions?

Reviewer #1: Partly

Reviewer #2: Yes

**********

3. Has the statistical analysis been performed appropriately and rigorously?

Reviewer #1: N/A

Reviewer #2: Yes

**********

4. Have the authors made all data underlying the findings in their manuscript fully available?

The PLOS Data policy

Reviewer #1: No

Reviewer #2: Yes

**********

5. Is the manuscript presented in an intelligible fashion and written in standard English?

Reviewer #1: No

Reviewer #2: Yes

**********

Reviewer #1: Thank you for submitting the revised manuscript entitled “Enjoy Life LocallY (ELLY): A qualitative study of a co-designed healthy weight and wellbeing intervention in two disadvantaged Scottish communities.”

The manuscript has been substantially improved in response to the previous review. In particular, the authors have strengthened the description of the qualitative methodology, participant recruitment and sampling, researcher reflexivity, analytical procedures, ethical considerations, and study limitations. The incorporation of additional participant perspectives, including less positive experiences, also improves the credibility and balance of the findings. The inclusion of the COREQ checklist, interview topic guide, and additional quotations as supporting information is also appreciated.

Overall, the manuscript is now considerably stronger and addresses most of the substantive concerns raised previously. I believe the study has potential to make a useful contribution to the literature on community co-designed interventions for disadvantaged populations. However, a few issues should be addressed before the manuscript can be considered further.

Major Comments

1. Please ensure consistency in the interpretation of qualitative findings and avoid causal language.

The revised manuscript appropriately acknowledges that qualitative data cannot establish definitive causation. However, some statements in the Abstract, Discussion and Conclusion remain stronger than the study design permits. For example, expressions such as “generated important and wide-ranging health outcomes”, “main drivers”, and “secondary consequence” may imply that causal relationships have been demonstrated.

Since the present paper is based on interviews with 20 participants, I recommend consistently framing these relationships as participant-reported experiences, perceived effects, or proposed pathways. For example, “participants perceived that…”, “participants described…”, “the findings suggest a potential pathway…”, or “these findings support the hypothesis that…” would be more appropriate than causal formulations.

2. Please revise the wording concerning “health improvements” in the Abstract.

The Abstract refers to “measurable health improvements”. However, this manuscript primarily presents qualitative accounts of participants’ experiences and perceptions. Unless objective outcome data are directly analysed and reported in this paper, “participant-reported health improvements” or “perceived improvements in health and wellbeing” would be more accurate.

3. Please reconsider the wording of the Conclusion regarding healthy weight.

The Conclusion currently suggests that wellbeing and social connection were the “main drivers” of behavioural change and that healthy weight was a “secondary consequence” of increased engagement and healthier routines.

This is an interesting interpretation and may represent an important hypothesis generated by the qualitative findings. However, the present study cannot establish that these factors caused changes in weight. I suggest reframing this as a proposed or perceived pathway. This would also make the Conclusion fully consistent with the authors’ own acknowledgement of the limitations of qualitative causal inference.

4. Please clarify the conceptual positioning of “snowball recruitment”.

In the Results, snowball recruitment is described as an “immediate outcome” of the intervention.

Recruitment and dissemination may be better conceptualised as an implementation/reach mechanism or an intermediate process rather than an intervention outcome. Please consider revising this terminology and explaining more clearly how snowball recruitment fits within the intervention logic model.

5. Please check the presentation of all figures carefully.

The conceptual figures are potentially an important contribution of the manuscript, but in the submitted reviewer version several figures appear cropped or truncated, particularly Figures 2–9, with portions of the right-hand side and some pathways not fully visible. Figure 10 also appears to extend beyond the displayed page area.

Please ensure that the final figure files are complete, sufficiently high resolution, and that all boxes, arrows, labels and outcome pathways are fully visible. The relationship between the individual figures and the final conceptual framework should also be clear to the reader.

Minor Comments

6. Please undertake a final English-language edit.

Although the revised manuscript is much improved, several grammatical and typographical problems remain. For example, there are instances of concatenated words, awkward sentence construction, repetition and inconsistent phrasing in the Introduction and elsewhere.

A thorough final language edit would substantially improve the readability and presentation of the manuscript.

7. Please review terminology relating to outcomes.

Terms such as “health improvement”, “weight loss”, “healthier diet”, “behaviour change” and “wellbeing” should be used consistently and, where appropriate, clearly distinguished between objectively measured outcomes and participant-reported experiences.

8. Please be cautious when describing the transferability of the findings.

The revised manuscript appropriately acknowledges the small qualitative sample and contextual nature of the study. Nevertheless, statements suggesting that the findings represent the wider population should be avoided or appropriately qualified. The experiences of the 20 interviewed participants should be understood within the specific contexts of the two participating communities.

9. Please further clarify the possibility of positive-selection bias among interview participants.

Although the revised manuscript provides a rationale for purposive selection of interview participants, participants who agreed to or were selected for interview may have differed from residents who engaged less with the intervention or withdrew. This possibility is partly acknowledged in the limitations but could be stated more explicitly.

10. Please undertake a final reference-format check.

The reference list has been substantially improved; however, some entries appear to contain inconsistencies between the “Available from” URL and DOI information, as well as minor formatting issues.

Please check all references for consistency with the journal’s reference style, particularly DOI and URL presentation.

Overall Comments

The revised manuscript represents a substantial improvement and most of the concerns raised in the previous review have been satisfactorily addressed. The study is potentially suitable for publication after minor revision.

I recommend Minor Revision, with particular attention to:

- avoiding causal overinterpretation of qualitative findings

- revising the wording of the Abstract and Conclusion accordingly

- clarifying the conceptual status of snowball recruitment

- ensuring that all figures are complete and fully legible, and

- undertaking a final language and reference-format check.

Reviewer #2: (No Response)

**********

what does this mean?). If published, this will include your full peer review and any attached files.

If you choose “no”, your identity will remain anonymous but your review may still be made public.

**Do you want your identity to be public for this peer review?**  For information about this choice, including consent withdrawal, please see our Privacy Policy

Reviewer #1: **Yes:**  Dr Anwar Basha, Scientist, ICMR-National Institute of Nutrition, Hyderabad (India).

Reviewer #2: No

**********

[NOTE: If reviewer comments were submitted as an attachment file, they will be attached to this email and accessible via the submission site. Please log into your account, locate the manuscript record, and check for the action link "View Attachments". If this link does not appear, there are no attachment files.]

To ensure your figures meet our technical requirements, please review our figure guidelines: https://journals.plos.org/plosone/s/figures

You may also use PLOS’s free figure tool, NAAS, to help you prepare publication quality figures: https://journals.plos.org/plosone/s/figures#loc-tools-for-figure-preparation.

NAAS will assess whether your figures meet our technical requirements by comparing each figure against our figure specifications.

# Author comment: Author response to Decision Letter 2

**DOI:** 10.1371/journal.pone.0361989.r004

2 Sep 2026

Reviewer 1: MINOR REVISION

Thank you for the very helpful comments provided by Reviewer 1. Below we have detailed points raised and how these have been addressed.

Reviewer 1 report comments

1. Please ensure consistency in the interpretation of qualitative findings and avoid causal language.

Thank you for highlighting these inconsistencies and causal language. We have now ensured language used is more appropriate and does not overstate relationships between what was reported and outcomes observed. Specifically:

Abstract

Line 35-36 we have replaced causal language with “support the hypothesis” and “associated with a range of important health outcomes”

Discussion

Line 494 “perceived causal pathways” added.

Line 500 “not perceived by participants as a motivating factor for changing behaviour” replaces the primary driver of behaviour change for participants within this population group.

Lines 503-4 “felt by participants” replaces reported by participants.

Lines 507-10 “felt by participants suggested” added; main driver replaced with “reason for taking part”; weight as a secondary outcome replaced with “potentially may reduce weight through”

Line 515 replaced ELLY can with “ELLY may”.

Lines 518-19 replaced achieved from being involved with “which participants felt was as a result of taking part in ELLY”.

Lines 534-35 replaced Our study showed with “Participants in our study reported”.

Lines 527-576 small amendments to language to avoid causal terminology.

Conclusions

Lines 596-600 replaced Findings show with “Findings suggest”; replaced main drivers with “associated with”; replaced secondary consequence with “potentially reflecting”.

2. Please revise the wording concerning “health improvements” in the Abstract

The wording in the Abstract has been revised to “participant-reported health improvements”, as suggested (Line 33).

3. Please reconsider the wording of the Conclusion regarding healthy weight.

Please see above changes to the Conclusion under Comment 1 above. In addition, wording has been amended from potential pathway to “how participants perceived ELLY had supported healthy weight and wellbeing” (line 597-8).

4. Please clarify the conceptual positioning of “snowball recruitment” We thank the reviewer for raising this important point about terminology. On reflection, we agree that snowball recruitment, and 'connecting with community assets' are better conceptualised as mechanisms through which the intervention operated, rather than as outcomes. The manuscript has been updated accordingly. Specifically:

Line 237 Table 2 has been updated to reflect these components as “mechanisms”.

Lines 240-41 replaced outcome with “intervention mechanism”.

Lines 256-60 Clarifies that the component is not a standalone outcome but rather a “reach and dissemination” pathway.

Line 259 Descriptor of Fig 2 updated to “Snowball recruitment as a reach and dissemination mechanism”.

Lines 284-86 Clarified component as a mechanism.

Line 287 Descriptor of Fig 3 updated to “Connecting with community assets awareness and engagement mechanism”.

Conclusion

Lines 485-87 Added “The model distinguishes intervention mechanisms, such as snowball recruitment and community connections, from the outcomes they contributed to, such as social connectedness and positive behaviour change.”

5. Please check the presentation of all figures carefully

The figures now all align with the PLOS One figure requirements which has improved visibility of all diagrams. More detail about the relationship between the figures and the conceptual model has been added.

6. Please undertake a final English-language edit.

A final edit has been completed to improve readability and presentation of the manuscript.

7. Please review terminology relating to outcomes.

The manuscript has been reviewed to clearly distinguish between participant reported and objectively measured outcomes.

8. Please be cautious when describing the transferability of the findings.

We have amended the language used in the Conclusions section of this paper accordingly, so as not to overstate the generalisability of our findings.

Specifically:

Line 610 Replaced generalisability with “relevance” which feels more appropriate.

Line 612-13 added “whether and how it may contribute”

Lines 612-19 changed the language to reflect the study providing “important insights”, “advancing understanding of how community interventions may support health and wellbeing and informing future research in the area”.

9. Please further clarify the possibility of positive-selection bias among interview participants.

As described in Lines 162-66 in Section ‘Qualitative data collection’, diversity of participants’ engagement in the intervention was achieved through purposive sampling. We have detailed the selection bias (not including those that dropped out of the study) with greater clarity in lines 603-06: “Although interviewees represented 27% of all participants and included participants with varying levels of engagement in the intervention, the sample did not include participants that dropped out of the study (n=9) who may have provided important insights that could have challenged or counterbalanced the findings”.

10. Please undertake a final reference-format check.

As per PLOS reference guidelines (Submission Guidelines | PLOS One) the “Available from” and URLs have been removed from published articles leaving only the full DOI.

Attachment Submitted filename: Response to Reviewers.docx

# Peer review: Decision Letter 2

**DOI:** 10.1371/journal.pone.0361989.r005

**Authors:** Rachel Suet Kay Chan

20 Oct 2026

Enjoy Life LocallY (ELLY): A qualitative study of a co-designed healthy weight and wellbeing intervention in two disadvantaged Scottish communities

PONE-D-26-07224R2

Dear Dr. Cowie,

We’re pleased to inform you that your manuscript has been judged scientifically suitable for publication and will be formally accepted for publication once it meets all outstanding technical requirements.

Within one week, you’ll receive an e-mail detailing the required amendments. When these have been addressed, you’ll receive a formal acceptance letter and your manuscript will be scheduled for publication.

An invoice will be generated when your article is formally accepted. Please note, if your institution has a publishing partnership with PLOS and your article meets the relevant criteria, all or part of your publication costs will be covered. Please make sure your user information is up-to-date by logging into Editorial Manager at Editorial Manager® and clicking the ‘Update My Information' link at the top of the page. For questions related to billing, please contact billing support.

If your institution or institutions have a press office, please notify them about your upcoming paper to help maximize its impact. If they’ll be preparing press materials, please inform our press team as soon as possible -- no later than 48 hours after receiving the formal acceptance. Your manuscript will remain under strict press embargo until 2 pm Eastern Time on the date of publication. For more information, please contact onepress@plos.org.

Kind regards,

Rachel Suet Kay Chan, Ph.D.

Academic Editor

PLOS One

Additional Editor Comments (optional):

Reviewers' comments:

Reviewer's Responses to Questions

**Comments to the Author**

Reviewer #1: (No Response)

**********

2. Is the manuscript technically sound, and do the data support the conclusions?

Reviewer #1: Yes

**********

3. Has the statistical analysis been performed appropriately and rigorously?

Reviewer #1: N/A

**********

4. Have the authors made all data underlying the findings in their manuscript fully available?

The PLOS Data policy

Reviewer #1: No

**********

5. Is the manuscript presented in an intelligible fashion and written in standard English?

Reviewer #1: No

**********

Reviewer #1: The authors have substantially revised the manuscript and addressed most concerns raised in the previous review. In particular, they have satisfactorily improved the qualitative methodology, reflexivity, sampling description, conceptual organization, reporting of limitations, distinction between mechanisms and outcomes, and supplementary materials.

However, several relatively minor issues remain and should be corrected before the manuscript can be considered for acceptance:

1. Residual causal language: Despite the authors' response that causal language has been removed, several statements still imply causation, including wording such as “how ELLY led to healthy weight and wellbeing outcomes” and “acted as a catalyst for further behaviour change.” These should consistently be reframed as participants' perceptions or reported associations.

2. Healthy weight/BMI interpretation: The discussion still appears to infer a pathway from the qualitative findings to weight/BMI outcomes. Because this study is qualitative and the BMI findings come from the accompanying quantitative study, clearly distinguish between the two.

3. Representativeness: The claim that the 20 interview participants are “representative of the wider population of ELLY feasibility study participants” seems stronger than the purposive sampling approach warrants. The authors should either support this with an explicit comparison or soften the claim.

4. Data Availability Statement: The submission information appears to indicate that all data are fully available without restriction, whereas the manuscript states that interview transcripts are subject to ethical/legal restrictions and are available upon request. These statements should be reconciled.

5. References: A few bibliographic inconsistencies remain despite the authors' statement that the references have been checked. In particular, verify and correct the DOI/link associated with References 4 and 15. Some “Available from:” entries also remain.

6. Final proofreading: The manuscript still contains several obvious typographical/grammatical errors, suggesting that the claimed final English edit should be checked once more.

**********

what does this mean?). If published, this will include your full peer review and any attached files.

If you choose “no”, your identity will remain anonymous but your review may still be made public.

**Do you want your identity to be public for this peer review?**  For information about this choice, including consent withdrawal, please see our Privacy Policy

Reviewer #1: **Yes:**  Dr D Anwar Basha, Scientist, ICMR-National Institute of Nutrition, Hyderabad, India

**********

# Editor report: Acceptance letter

**DOI:** 10.1371/journal.pone.0361989.r006

**Authors:** Rachel Chan

PONE-D-26-07224R2

PLOS One

Dear Dr. Cowie,

I'm pleased to inform you that your manuscript has been deemed suitable for publication in PLOS One. Congratulations! Your manuscript is now being handed over to our production team.

At this stage, our production department will prepare your paper for publication. This includes ensuring the following:

* All references, tables, and figures are properly cited

* All relevant supporting information is included in the manuscript submission,

* There are no issues that prevent the paper from being properly typeset

You will receive further instructions from the production team, including instructions on how to review your proof when it is ready. Please keep in mind that we are working through a large volume of accepted articles, so please give us a few days to review your paper and let you know the next and final steps.

Lastly, if your institution or institutions have a press office, please let them know about your upcoming paper now to help maximize its impact. If they'll be preparing press materials, please inform our press team within the next 48 hours. Your manuscript will remain under strict press embargo until 2 pm Eastern Time on the date of publication. For more information, please contact onepress@plos.org.

You will receive an invoice from PLOS for your publication fee after your manuscript has reached the completed accept phase. If you receive an email requesting payment before acceptance or for any other service, this may be a phishing scheme. Learn how to identify phishing emails and protect your accounts at https://explore.plos.org/phishing.

If we can help with anything else, please email us at customercare@plos.org.

Thank you for submitting your work to PLOS One and supporting open access.

Kind regards,

PLOS One Editorial Office Staff

on behalf of

Dr. Rachel Suet Kay Chan

Academic Editor

PLOS One
